# Comparative Analysis on Proteomics Profiles of Intracellular and Extracellular *M.tb* and BCG From Infected Human Macrophages

**DOI:** 10.3389/fgene.2022.847838

**Published:** 2022-03-28

**Authors:** Han Liu, Li Su, Tingting Zhu, Xiaojie Zhu, Yifan Zhu, Yonchong Peng, Kailun Zhang, Longwei Wang, Changmin Hu, Huanchun Chen, Yingyu Chen, Aizhen Guo

**Affiliations:** ^1^ The State Key Laboratory of Agricultural Microbiology, Huazhong Agricultural University, Wuhan, China; ^2^ College of Veterinary Medicine, Huazhong Agricultural University, Wuhan, China; ^3^ National Animal Tuberculosis Para-Reference Laboratory (Wuhan) of Ministry of Agriculture and Rural Affairs, Huazhong Agricultural University, Wuhan, China; ^4^ Hubei International Scientific and Technological Cooperation Base of Veterinary Epidemiology, Huazhong Agricultural University, Wuhan, China

**Keywords:** tuberculosis, *M.tb*, BCG, proteomics, macrophages, virulence

## Abstract

Tuberculosis is the second cause in infectious diseases leading to human death. Understanding the virulence mechanism is inevitable if the disease needs to be fully cured. Therefore, this study aimed to reveal this mechanism by comparing proteomic profiles of intracellular and extracellular virulent strain *M.tb* and bacille Calmette–Guérin (BCG) from infected THP-1cells. First, *M.tb* and BCG infected THP-1 at MOI 10:1. Twelve hours postinfection, intracellular bacteria of *M.tb* and BCG were collected, whereas the two bacilli cultured in 7H9 broth media were used as the control. Then four groups of bacilli were subjected to proteomic analysis, and differential proteomic profiles between *M.tb* and BCG were comparatively analyzed with bioinformatics tools. As a result, we identified a total of 1,557 proteins. Further, they were divided into four groups for comparison of *M.tb* versus BCG under 7H9 culture (shorten as out), *M.tb* in (intracellular) versus *M.tb* out, BCG in versus BCG out and *M.tb* in versus BCG in. Between *M.tb* in versus BCG in, a total of 211 differentially expressed proteins were found. Eight proteins like ESAT-6 distributed in six RDs and some known proteins related to virulence. Besides, five uncharacterized proteins were differentially expressed. Further analysis revealed enriched pathways were associated with glyoxylate and dicarboxylate metabolism pathways. In *M.tb* out versus BCG out, a total of 144 differential proteins were identified and mainly involved in metabolism pathways. Then, 121 differential proteins in the group of *M.tb* in versus *M.tb* out were enriched in ribosome and oxidative phosphorylation related to adaptation to the host environment. The group of BCG in versus BCG out shared the same trend of different pathways to the *M.tb* in versus *M.tb* out. Finally, 42 proteins were identified to be up-regulated only in intracellular *M.tb* including eight RD proteins, whereas 22 up-regulated uniquely in intracellular BCG. Besides, only two proteins (Pks13 and Rv1405c) were commonly up-regulated in intracellular *M.tb* and BCG. Further, some unknown proteins were uniquely up-regulated in the intracellular *M.tb* and BCG. These findings provide valuable data for further exploration of molecular mechanism for *M.tb* virulence and BCG immune response.

## Introduction

*Mycobacterium tuberculosis* (*M.tb*), the etiologic agent of tuberculosis (TB), remains a serious global public health threat. As an extremely successful intracellular bacteria, *M.tb* can survive and cause disease correlated to their unique immune evasion mechanism. Despite the availability of anti-TB drugs, the normal treatment time lasts 6 to 12 months. In addition, increasing multidrug-resistant TB and extensively drug-resistant TB are bringing great difficulties to realize End-TB strategy proposed by the World Health Organization (Cohen et al., 2019).

*Mycobacterium bovis* bacille Calmette–Guérin (BCG), an attenuated strain of *M. bovis* that developed more than a century ago and remains the only licensed TB vaccine in humans for 100 years. Although the efficacy of BCG against active TB in adults has been challenged, it saves millions of lives by preventing severe, miliary, and meningeal TB, especially for children (Zhu et al., 2018). Genomic comparisons reveal at least 18 variable regions of difference (named RDs) representing more than 100 genes are deleted in BCG but present in *M.tb*. For example, the RD1 region contains ESAT-6 system and PE and PPE proteins, whereas the RD2 region includes MPT6, transcriptional regulator, and others (Brosch et al., 2001).

*M.tb* is an intracellular pathogen and mainly resides in macrophages and dendritic cells. To establish an infection, *M.tb* should internalize the host cells, survive, and reproduce in the environment of phagosomes with low pH, oxygen, and starvation. Further, the bacteria should win the fight of host defense called innate immunity including inflammatory responses (Liu et al., 2017), antimicrobial peptides, toxic antimicrobial effectors such as nitric oxide, or reactive oxygen intermediates in macrophages (Weiss and Schaible, 2015).

With coevolution of *M.tb* and the hosts, *M.tb* has evolved a series of strategies to evade immune responses and remains persistent infection for a long term in the hosts. For instance, *M.tb* is capable of recruiting GTPase Rab5 from phagosomes to inhibit phagolysosome maturation (Carranza and Chavez-Galan, 2019). In addition, *M.tb* can produce lots of 1-tuberculosinyladenosine and be selectively accumulated in the host cells to change its cellular microenvironment as a bacterial antacid after infection. This bacillus can utilize the host’s ubiquitination system to suppress host immune response (Wang et al., 2020) and mediate cellular events such as apoptosis and autophagy via various approaches such as miRNA (Li et al., 2019; Zhao et al., 2019). Evasion of TLR or NLR signaling and C-type lectin receptor signaling also help *M.tb* to resist the host innate immunity (Goldberg et al., 2014).

However, up to now, the virulence mechanism of *M.tb* is far away from complete understanding. To reveal the differential proteomic profiles between intracellular *M.tb* and BCG under infection would reveal the new mechanism to understand the virulence of *M.tb* and TB pathogenesis.

In this article, by using a model of macrophages infected with virulent *M.tb* and attenuated strains BCG, we compared the different proteomics of intracellular *M.tb* and BCG by using the cultured bacteria as the controls. The enriched differential pathways and individual proteins related to virulence would provide evidence for further elucidation of *M.tb* virulence and TB pathogenesis.

## Methods

### Bacterial Strains and Cell Culture

*M.tb* 1,458 strain (GenBank accession no. NZ_CP013475) is a strain of bovine origin isolated by the National Animal Tuberculosis Para-Reference Laboratory (Wuhan) at Huazhong agricultural university, Wuhan, China (Xiong et al., 2017). BCG Tokyo 172 (ATCC 35737, GenBank accession no.GCA_000010685.1) was a kind gift from Prof. Junyan Liu (School of Basic Medicine Sciences, Wuhan University, China). Both strains were grown in broth medium Middlebrook 7H9 with 10% of oleic acid–albumin–dextrose–catalase (BD, Franklin, NJ, United States), 0.5% glycerol (Sigma–Aldrich), and 0.05% Tween 80 (Sigma–Aldrich) at 37°C. The human acute monocytic leukemia cell line THP-1 (ATCC TIB-202) was cultured in RPMI 1640 medium (HyClone, United States) containing 10% fetal bovine serum (Gibco, United States) with 5% CO_2_ at 37°C.

### Infection Model of THP-1 Cells

THP-1 cells were seeded at 30 × 10^6^ cells/well in a 145-mm cell culture dish and differentiated with 40 ng/ml phorbol 12-myristate 13-acetate (PMA) (Sigma-Aldrich, St. Louis, United States) for 12 h, at 37°C. For infection, the two logarithmic growing cultures (*M.tb* and BCG) were centrifuged and resuspended with Hanks balanced salt solution (HBSS) (Gibco). The bacterial concentrations (colony-forming units/ml) were estimated by measuring the OD_600_ values and infected at MOI (BCG, or MTB-1458: THP-1cells) of 10:1. After 12 h, the extracellular bacteria in the culture medium were collected. The cells were washed twice with RPMI 1640 fresh medium and incubated with a fresh RPMI 1640 medium containing 100 µg/ml gentamicin to kill extracellular bacteria.

The bacteria were confirmed by acid-fast staining. Briefly, the smears of infected THP-1 cells were permeabilized with 0.4% triton X-100 for 10 min and washed twice with phosphate-buffered saline. Finally, the acid-fast staining was performed using acid-fast staining solution kit (Yuan Ye Biological Technology, Shanghai, China) according to the manufacturer’s instruction.

### Extraction of Bacterial Proteomics and iTRAQ Labeling

For intracellular bacterial proteins, the infected cells were lysed with radioimmunoprecipitation assay (RIPA) lysis buffer (Sigma-Aldrich, St. Louis, United States) containing protease and phosphatase inhibitors (Roche, Basel, Switzerland) and subjected to be centrifuged for 15,000*g* for 2 min at 4°C. The pellet was collected and added 1 ml 1% sodium dodecyl sulfate (SDS) for resuspension.

Bacteria growing to logarithmic phase were centrifuged at 4,000*g* for 10 min. The pelleted washed by HBSS and centrifuged at 4,000*g* for 10 min. Then, 1 ml HBSS was resuspended, pelleted, and further lysed with sonication. The parameters were set as 1 s/1-s intervals, 3-min time, and 80-W power. After sonication, the samples were centrifuged at 15,000*g* for 15 min to remove insoluble particles, repeated once to further exclude precipitation. The bacterial proteins that contained membrane proteins were collected, and the concentration was determined by BCA protein assay kit (Beyotime). Finally, the sample was stored at −80°C for the following steps.

For tryptic digestion and iTRAQ labeling, 50 µg of each protein sample was mixed with 120 µl reduction buffer (10 mM DTT, 8 M urea, 100 mM triethylammonium bicarbonate (TEAB), pH 8.0) on a 10 K ultrafiltration tube, and incubated at 60°C for 1 h, and then IAA (3-indoleacetic acid) was added at a final concentration of 50 mM and stayed at room temperature for 40 min under protection from light. The solution was then centrifuged on the filter at 12,000*g* for 20 min at 4°C and the concentrated protein solution on the filter was added 100 µL of TEAB (100 mM) and centrifuged at 12,000*g* for 20 min. Then 100 µl of 100 mM TEAB and 2 µl trypsin (1 µg/µl) were added to each tube and then incubated at 37°C for 12 h. Finally, the digested peptides were centrifuged at 12,000*g* for 20 min, 50 µl of 100 mM TEAB were added and repeated centrifugation again. The generated peptides were dried by vacuum centrifugation and re-dissolved with 100 µl of 0.5 mM TEAB. The 40 µl of each sample were transferred into a new tube and 100 µl of the iTRAQ label reagent dissolved in isopropanol was added. Finally, 200 µl of high-performance liquid chromatography (HPLC) water were added and incubated for 30 min to terminate the reaction.

### Reversed-Phase Liquid Chromatographic and Mass Spectrometry Analysis

A 1100 HPLC system (Agilent) was used for reversed-phase (RP) chromatographic separation with an Agilent Zorbax Extend RP column (5 µm, 150 × 2.1 mm). Mobile phases A (2% acetonitrile in HPLC water) and B (98% acetonitrile in HPLC water) were used for the RP gradient. Trypsin peptides were separated at a steady flow rate of 300 µl/min, monitored at 210 and 280 nm. Then elution buffer at every minute was collected, lasting for 8–50 min. The separated peptides were lyophilized for mass spectrometer (MS) detection as follows.

All analyses were performed by a Q-Exactive MS (Thermo, United States) equipped with a nanospray flex source (Thermo, United States). The peptides were separated on a C18 analytical RP column (75 µm × 15 cm) at a flow rate of 300 nL/min and a linear gradient of 70 min. A full MS scan was obtained in the mass range of 300 to 1600 m/z. Ten strongest peaks in the MS were fragmented by high-energy collision dissociation. The resolution of the obtained MS/MS spectrum is 17,500 with the maximum injection time of 50 ms. Q-E dynamic exclusion was set to 15 s.

All of the Q exactive MS/MS raw data were analyzed by using proteome discoverer v.2.2 (Thermo Company, United States) software and the uniprot-*M.tb* database. The false-positive rate of peptide identification was controlled below 1%.

### Bioinformatics Analysis

To compare the proteomics with 1,557 proteins, we divided them into four groups. Among them, the intracellular bacteria of *M.tb* and BCG from the macrophages were defined *M.tb* in and BCG in, whereas the extracellular bacteria cultured in 7H9 medium were defined *M.tb* out and BCG out. So, the four groups are BCG in versus *M.tb* in, BCG out versus *M.tb* out, *M.tb* in vs. *M.tb* out, and BCG in vs. BCG out.

Database searches were performed for proteins analyzed by RP liquid chromatography and MS. Experimental data were analyzed by Proteome Discoverer™ 2.2 (Thermo, United States) software. The data are from the Uniprot–*Mycobacterium tuberculosis* database. For the obtained search results, only unique peptides were contained for iTRAQ labeling quantification, and peptides with global false discovery rate (FDR) values from fit less than 1% were considered for further analysis. Based on the screened credible proteins, the difference multiple fold change (FC) value and difference significance *p* value of each comparison group were calculated. The proteins with significant differences were screened according to the criteria of FC >1.5 or FC < 2/3 and *p* < 0.05. Different proteins in four groups were analyzed by heatmaps and volcano plots based on a threshold FC >1.5 and *p*-value < 0.05. Using the OmicsBean omics data integration analysis cloud platform (http://www.omicsbean.cn/), the Kyoto Encyclopedia of Genes and Genomes (KEGG) analysis was performed on the selected differential proteins. The KEGG analysis was used corrected *p*-value < 0.05 as a threshold for finding significantly enriched terms in the input list of differentially expressed proteins. To find the shared mechanism for both *M.tb* and BCG necessary mechanism to infection, the differential proteins with twofold difference were checked to analyze and identify commonly regulated proteins in different groups.

## Results

### Quantitative and Qualitative Evaluation of Protein Samples

**FIGURE 1:**
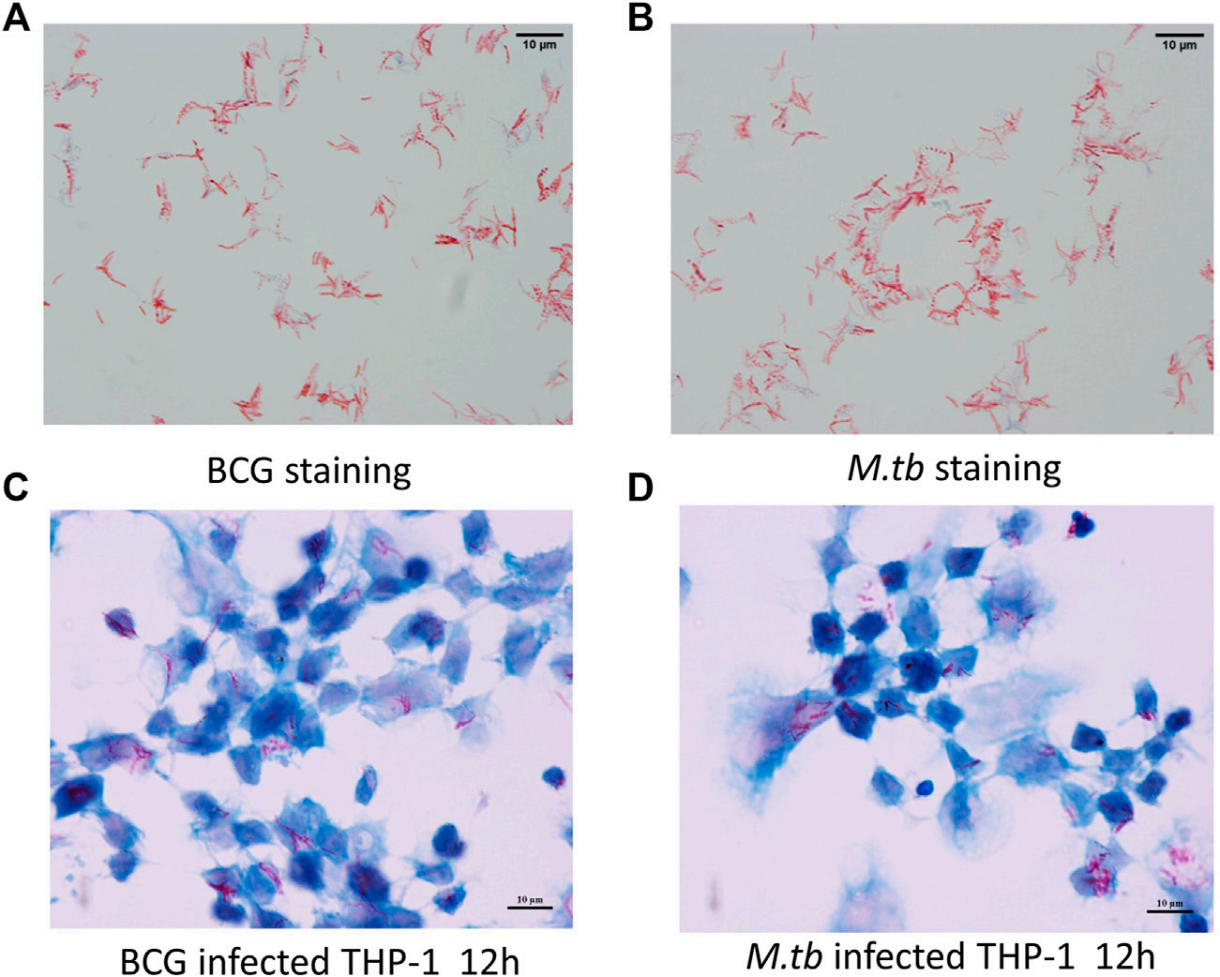
Infection model. PMA-differentiated THP-1 cells were infected with *M.tb* and BCG for 12 h. Ziehl–Neelsen staining of the BCG *in vitro* cultured **(A)** and *M.tb* **(B)**. Ziehl–Neelsen staining to detect the survival of BCG **(C)** and *M.tb* **(D)** in THP-1 cells.

By using fast-acid staining, the smear showed *M.tb* and BCG in red and THP-1 in blue, demonstrating the infection was successful (Figure 1).

**FIGURE 2:**
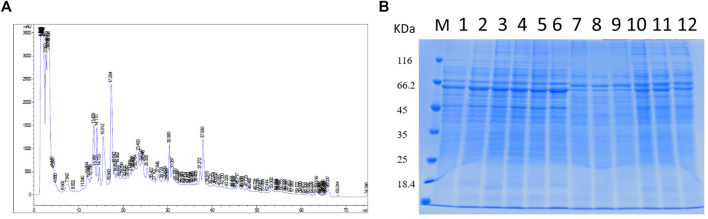
Quality control and overall primary results. **(A)** Extract the total protein in the sample and take out a part for protein concentration determination to SDS-PAGE detection. The molecular weights from top to bottom are as follows: 116, 66.2, 45,35, 25, and 18.4 (kDa). **(B)** High-pH RP chromatography separation peak diagram. The protein is trypsinized and labeled, and then the same amount of each labeled sample is mixed for chromatographic separation.1–12: BCG in-1, BCG in-2, BCG in-3, *M.tb* in-1, *M.tb* in-2, *M.tb* in-3, BCG1, BCG2, BCG3, *M.tb*1, *M.tb* 2, *M.tb* 3.

**FIGURE 3:**
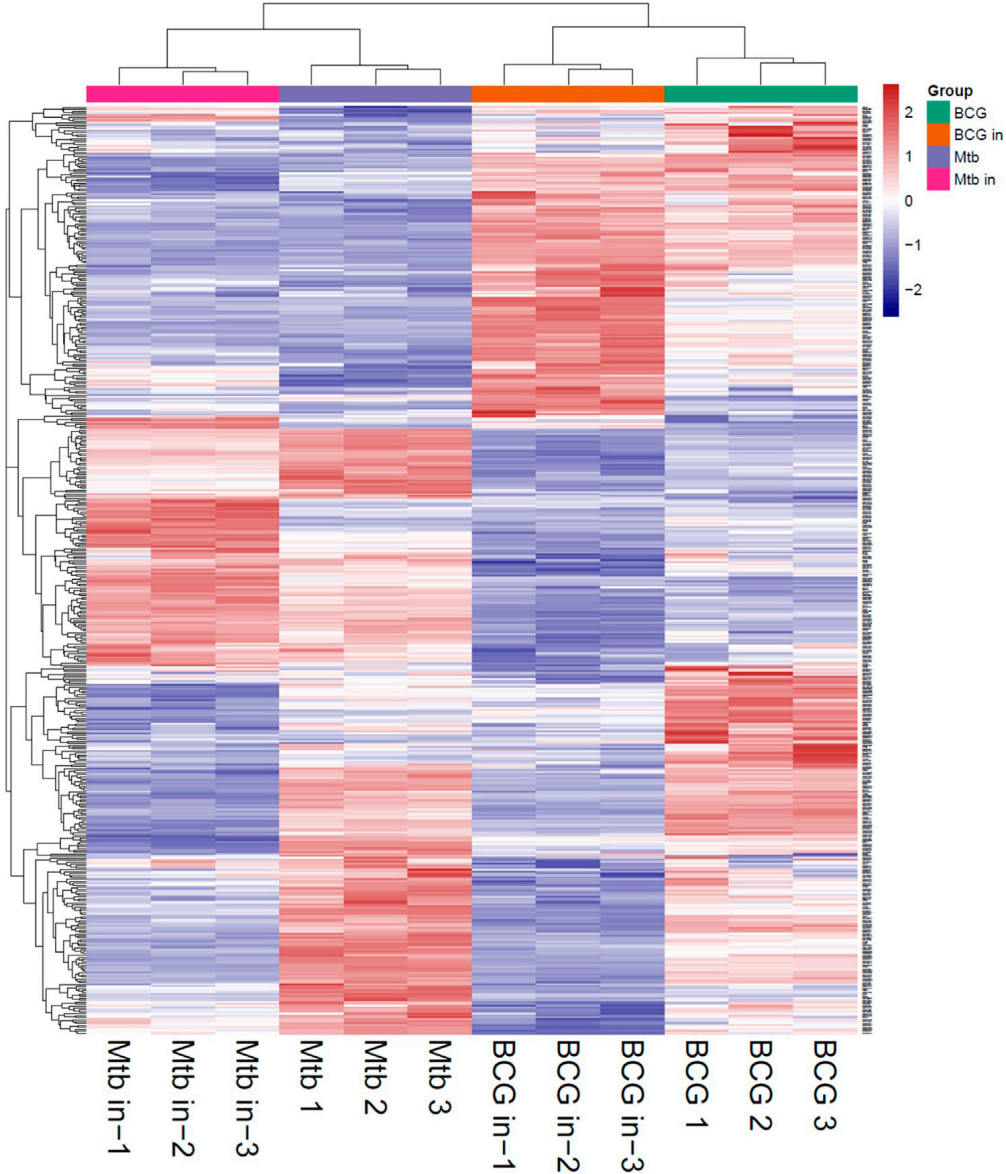
Heatmap of all groups (*M.tb*, intracellular *M.tb*, BCG, and intracellular BCG). Heatmap of proteins differentially expressed in all groups. Blue-to-red scale indicates low-to-high proteins expression levels. Differentially expressed proteins were defined based on a threshold fold change >1.5, at *p* < 0.05.

The proteins collected from the bacteria were analyzed by SDS–polyacrylamide gel electrophoresis (PAGE) and RP chromatography (Figure 2). Combining the two checking methods, the replicates from the same experiment showed the similar trend representing that the extraction methods were reliable for further analysis. In addition, Table 1 showed the sample concentrations were sufficient for analysis. First, all the MS data of four bacterial samples (BCG in and BCG out, *M.tb* in and *M.tb* out) each in triplicate numbering 1 to 3 were divided into three groups 1, 2, and 3 by pooling the same number of the group into one group first for analysis of qualitative proteins by base search. Then, the data were filtered with a global FDR < 0.01 to obtain quantitative protein 1 to 3. Finally, we identified 2,442, 2,625, and 2,584 valid proteins for groups 1 to 3 samples (Table 2; Supplementary Table S1). In the clustering analysis of the proteins according to different conditions revealed in the group of BCG in and *M.tb* in, 211 differentiated proteins were found, and half of them shared by BCG in and *M.tb* in, and another half were different. In the group of *M.tb* in and *M.tb* out, two-thirds of the total proteins were shared with similar change trend, whereas the remaining one-third proteins showed opposite trends. The same situation was found in the group of BCG in and BCG out. However, when proteomics of BCG out and *M.tb* out were compared, only one-third of the total proteins were shared with similar change trend, whereas the remaining two-thirds proteins showed opposite trends (Figure 3). Taken together, both the intracellular environment and virulence differences affected the expression of proteomics of BCG and *M.tb*.

**TABLE 1:** The absorbance and concentrations.

Number	Absorbance 1	Absorbance 2	Absorbance 3	Average absorbance	Concentration (µg/µL)
BCG in 1	0.086	0.079	0.08	0.082	0.793
BCG in 2	0.116	0.105	0.111	0.111	1.078
BCG in 3	0.201	0.187	0.187	0.192	1.874
*M.tb* in 1	0.25	0.243	0.241	0.245	2.394
*M.tb* in 2	0.097	0.089	0.091	0.092	0.898
*M.tb* in 3	0.106	0.099	0.098	0.101	0.983
BCG 1	0.071	0.064	0.065	0.067	0.646
BCG 2	0.062	0.059	0.055	0.059	0.567
BCG 3	0.079	0.069	0.068	0.072	0.698
*M.tb* 1	0.256	0.239	0.244	0.246	2.410
*M.tb* 2	0.323	0.303	0.311	0.312	3.059
*M.tb* 3	0.249	0.233	0.237	0.240	2.345

**TABLE 2:** The reliable numbers of the proteins identified.

Protein type	Qualitative protein 1	Quantitative protein 1	Qualitative protein 2	Quantitative protein 2	Qualitative protein 3	Quantitative protein 3
FDR <1%	2,634	2,442	2,828	2,625	2,797	2,584

### Differentiated Protein Expression Between BCG in and *M.tb* in During the Infection of Macrophages (BCG In vs. *M.tb* In)

**FIGURE 4:**
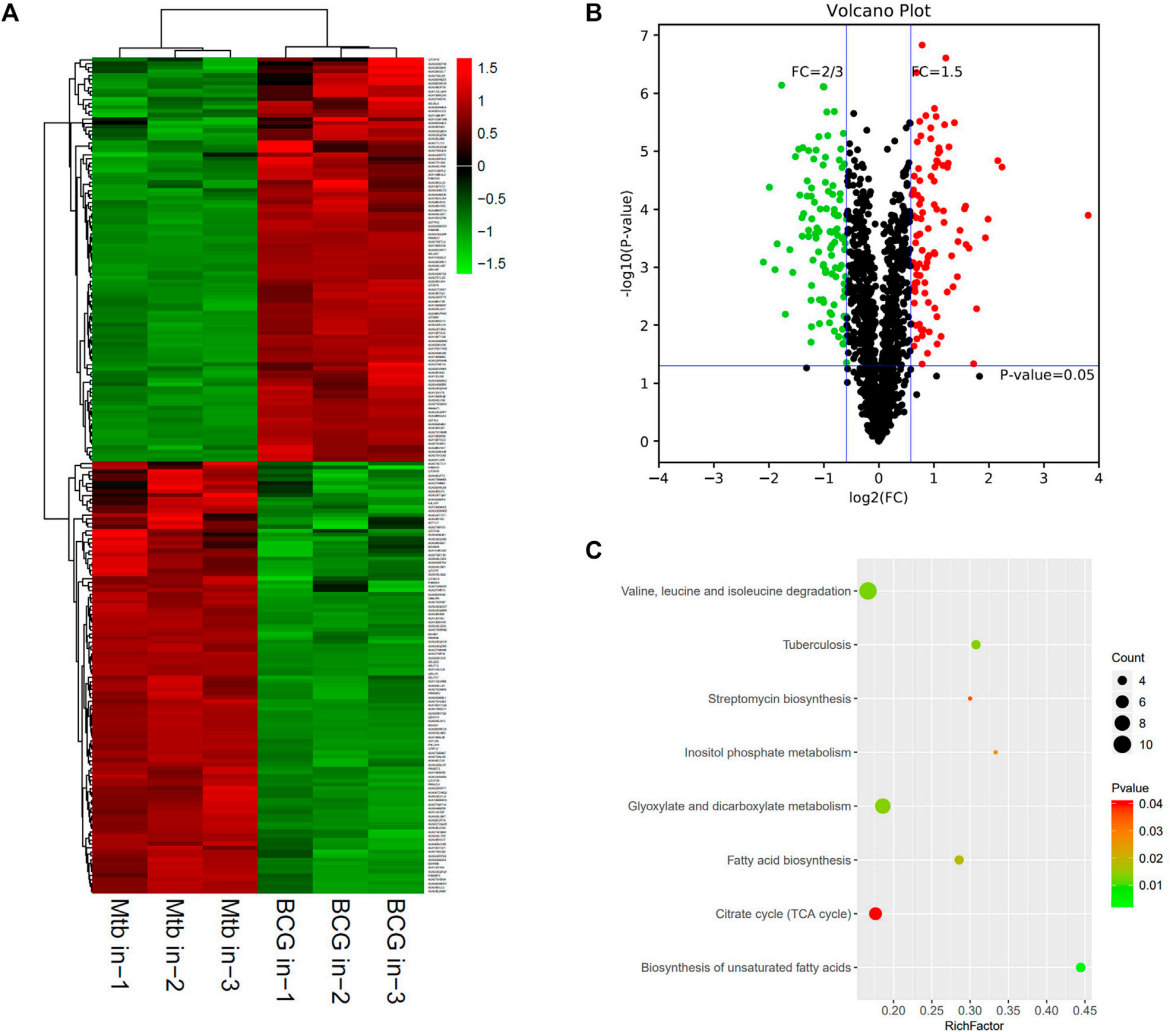
Different protein profile between intracellular BCG and intracellular *M.tb*. **(A)** Heatmap of proteins differentially expressed between the group of intracellular BCG and intracellular *M.tb*. Green-to-red scale indicates low-to-high proteins expression levels (*p* < 0.05). **(B)** Volcano plots of proteins differentially expressed between the group of intracellular BCG and intracellular *M.tb*. The red dots on the right indicate the significantly upregulated proteins, whereas the downregulated are shown with green dots on the left. Differentially expressed proteins were defined based on a threshold fold change >1.5, at *p* < 0.05. **(C)** Top10 KEGG pathways (only eight in this group) enriched for the significantly differentially expressed proteins. Entries with larger bubbles contain more differential genes. The color of the bubbles indicates the *p* value. The smaller the enrichment *p* value means the greater the degree of significance (*p* < 0.05).

The proteomic profiles of BCG in vs. *M.tb* in were comparatively analyzed. The dysregulated proteins total to 211 between the intracellular *M.tb* and BCG. Almost equal numbers of genes were down-regulated (109) and up-regulated (102). Compared with the group of BCG out and *M.tb* out, their gene expression profiles in this group showed more diversity (Figures 4A,B). The top 10 up-regulated proteins in BCG compared with *M.tb* include RpsA, FadD29, Rv1473, Rv3699, SseA, Rv1358, Rv2588c, Rv1762c, Pks13, and Rv0025; among them, RpsA, the 30 S ribosomal protein S1, was mostly up-regulated (13.9-fold up). Rv2588c, Rv1762c, and Rv0025 were the proteins uncharacterized (Supplementary Table S2). On the contrary, the top 10 down-regulated proteins in BCG compared with *M.tb* include LppE, Rv3099c, PncB2, Rv1498A, VapB19, PstS1, Rv0795, Rv1817, Gnd1, and TB22.2, among them, TB22.2 was mostly down-regulated (five folds down) (Table 3). TB22.2 (Rv3036c), as a surface-anchored esterase, may play a role in structurally modifying the cell wall composition when *M.tb* infection (Trivedi et al., 2005). In addition, of the top 10 down-regulated proteins in BCG compared with *M.tb*, some differential known proteins including PPE family (PPE41 and PPE51) and RD regions EspK (RD1), EsxA (RD1), EsxO (RD7), LpqG (RD9), Rv0223c (RD4), epiA (RD6), Rv2073c (RD12), and Rv1513 (RD6). The functional studies of Rv2073c, EpiA, and Rv1513 remain not clear and deserve further study.

**TABLE 3:** The top10 differentially expressed proteins between intracellular BCG and *M.tb*.

Genes	Fold change	UNIPROT_URL	Annotation	Differential expression
rpsA	13.94335938	http://www.uniprot.org/uniprot/P9WH43	rpsA–30S ribosomal protein S1	Up
fadD29	4.713255185	http://www.uniprot.org/uniprot/P95141	Long-chain fatty acid–AMP ligase FadD29	Up
Rv1473	4.471317829	http://www.uniprot.org/uniprot/O53164	Macrolide ABC transporter ATP-binding protein	Up
Rv3699	3.954741379	http://www.uniprot.org/uniprot/O69667	Hypothetical protein	Up
SseA	3.82406015	http://www.uniprot.org/uniprot/P9WHF7	Thiosulfate sulfurtransferase SseA	Up
Rv1358	3.421887391	http://www.uniprot.org/uniprot/Q11028	Transcriptional regulator	Up
Rv2588c	3.295882763	http://www.uniprot.org/uniprot/P9WL75	Membrane protein secretion factor YajC	Up
Rv1762c	3.112998041	http://www.uniprot.org/uniprot/O06797	Hypothetical protein	Up
pks13	2.997491219	http://www.uniprot.org/uniprot/I6X8D2	Polyketide synthase	Up
Rv0025	2.979821958	http://www.uniprot.org/uniprot/P9WMA1	Hypothetical protein	Up
lppE	0.369435344	http://www.uniprot.org/uniprot/O07750	Lipoprotein LppE	Down
Rv3099c	0.364488171	http://www.uniprot.org/uniprot/O05777	Hypothetical protein	Down
pncB2	0.349864499	http://www.uniprot.org/uniprot/P9WJI7	Nicotinic acid phosphoribosyltransferase PncB2	Down
Rv1498A	0.325813729	http://www.uniprot.org/uniprot/I6XY36	Hypothetical protein	Down
vapB19	0.308091531	http://www.uniprot.org/uniprot/P95006	Antitoxin VapB19	Down
pstS1	0.293881008	http://www.uniprot.org/uniprot/P9WGU1	Phosphate ABC transporter substrate–binding lipoprotein PstS	Down
Rv0795	0.277755018	http://www.uniprot.org/uniprot/P9WKH5	Hypothetical protein	Down
Rv1817	0.270431497	http://www.uniprot.org/uniprot/Q50616	Flavoprotein	Down
gnd1	0.251934327	http://www.uniprot.org/uniprot/Q79FJ2	6-Phosphogluconate dehydrogenase	Down
TB22.2	0.233486401	http://www.uniprot.org/uniprot/I6YF08	Cell wall–anchored esterase	Down

KEGG pathway of the differential expressed genes was further performed and showed that glyoxylate and dicarboxylate metabolism; valine, leucine, and isoleucine degradation; and TB are the top three pathways enriched (Figure 4C). Glyoxylate and dicarboxylate metabolism is essential for mycobacterial persistence; the enzymes of the glyoxylate cycle are activated during adaption to low oxygen environment. As glyoxylate and dicarboxylate metabolism was upregulated more in intracellular BCG than *M.tb*, it might be used by BCG to adapt to the environment within macrophages. On the other hand, the intracellular *M.tb* relies more on TB pathway than BCG shown by the differential proteins including ESAT-6 (esxA), a protein in RD1, pstS1, and lprA up-regulated, whereas pstS3 down-regulated in *M.tb* compared with BCG.

### Differential Proteomic Profiles Between BCG and *M.tb* in Medium Culture (BCG Out vs. *M.tb* Out)

**FIGURE 5:**
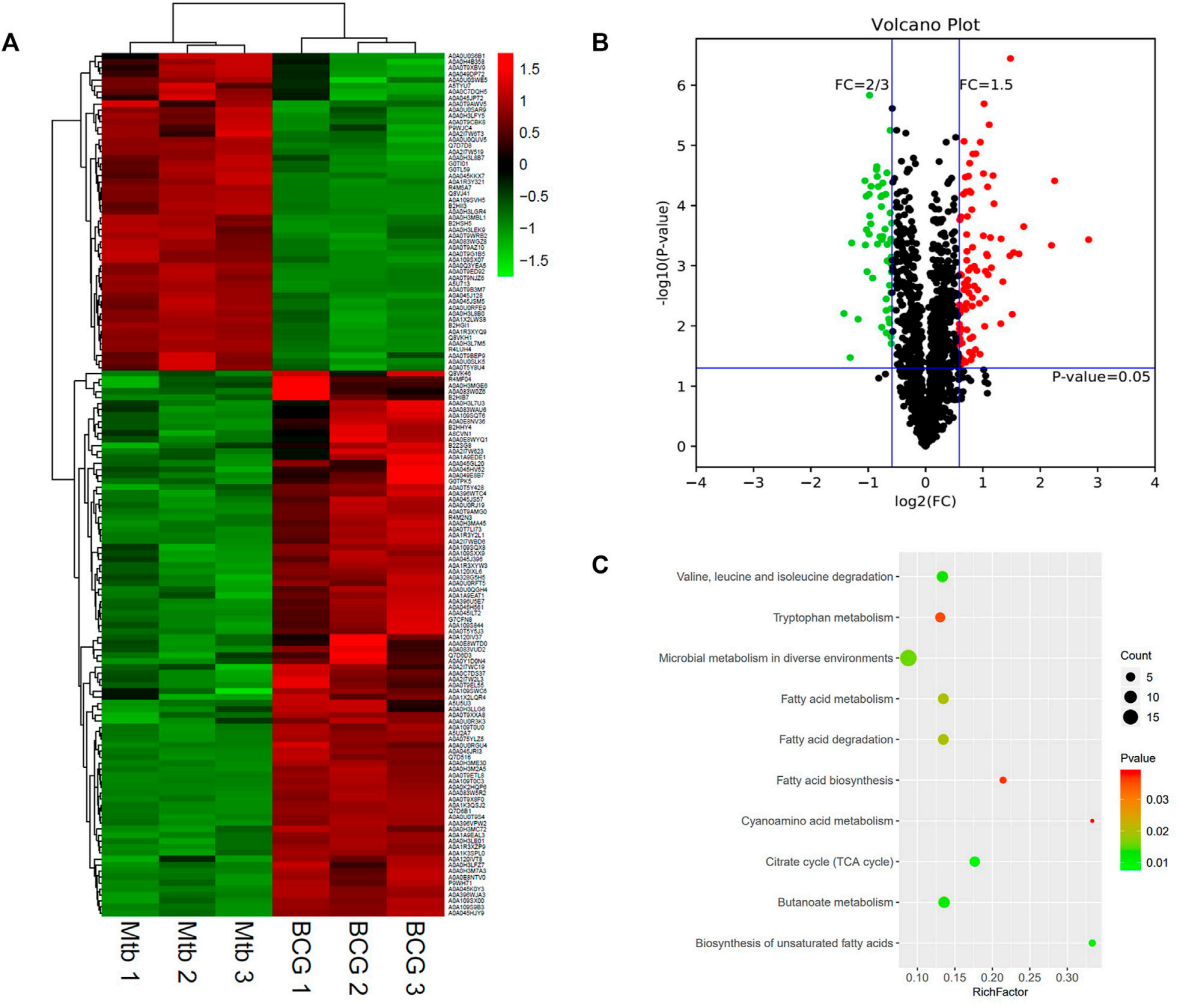
Differential protein profile between *M.tb* and BCG. **(A)** Heatmap of proteins differentially expressed between the group of *M.tb* and BCG. Green-to-red scale indicates low-to-high proteins expression levels (*p* < 0.05). **(B)** Volcano plots of proteins differentially expressed between the group of *M.tb* and BCG. The red dots on the right indicate the significantly upregulated proteins, whereas the downregulated proteins are shown with green dots on the left. Differentially expressed proteins were defined based on a threshold fold change >1.5, at *p* < 0.05. **(C)** The top 10 KEGG pathways enriched for the significantly differentially expressed proteins. Entries with larger bubbles contain more differential genes. The color of the bubbles indicates the *p* value. The smaller the enrichment *p* value means the greater the degree of significance (*p* < 0.05).

The differential proteomics between BCG out and *M.tb* out was analyzed. The cluster heatmap showed that both BCG and *M.tb* samples were clustered into two groups. In *M.tb*, there are more genes downregulated, whereas the trend was opposite in BCG samples (Figure 5A). Based on the trusted protein of *M.tb* selected, 91 proteins were upregulated and 53 downregulated in BCG (Figure 5B). The top 10 up-regulated proteins in BCG compared with *M.tb* include RpsA, FadD29, Rv1358, Rv3699, Rv1473, SseA, Rv3406, GltD, Ino1, and Pks13. Among them, RpsA was also the top1 up-regulation proteins, same as the group of *M.tb* in and BCG in (7.1-fold up). Rv3699 was uncharacterized, and Rv1358 was defined as transcriptional regulator, but the detailed functions remain unknown (Supplementary Table S2). On the contrary, the top 10 down-regulated proteins in BCG compared with *M.tb* include MmaA4,DesA1,PncB2,Rv0248c, Rv0247c, FadD15, Rv1817, HupB, FadD28, and Rv1514c; among them, Rv1514c was mostly down-regulated (two-point sevenfold down) (Table 4). For Rv1514c, the binding site prediction confirmed it might be bounded with UDP (uridine-diphosphate), and it could be a transferase activity protein, which possibly transfers glycosyl group action (Beg et al., 2018). It gives us the hint that *M.tb* needs more glycosyltransferase than BCG. Rv1817, relevant with oxidoreductase activity, was found dropped significantly in BCG compared with *M.tb*. The functions of genes Rv1358 and Rv3699 remain unknown in *M.tb* or BCG (Supplementary Table S2). In addition, of the top 10 proteins, Hsp and pstS3 were up-regulated in BCG compared with *M.tb* and play an important role in knowing the mechanism of protective immune responses against TB. It has been reported that heat shock protein (Hsp) generates production in low-virulence strains of mycobacteria to help persist in host macrophages and a crucial chaperon protein necessary for the survival of *M.tb* in the dormant phase (Dubaniewicz, 2010). Psts3 is a surface-exposed phosphate transport receptor. The use of DNA vaccine encoding pstS3 to trigger immune response can significantly protect mice attacked by *M.tb* (Tanghe et al., 1999).

**TABLE 4:** The top 10 differentially expressed proteins in BCG based on *M.tb.*

Genes	Fold change	UNIPROT_URL	Annotation	Differential expression
rpsA	7.17481203	http://www.uniprot.org/uniprot/P9WH43	30s ribosomal protein s1 rpsa	Up
fadD29	4.75609756	http://www.uniprot.org/uniprot/P95141	Fatty acid–CoA ligase	Up
Rv1358	4.58123371	http://www.uniprot.org/uniprot/Q11028	Uncharacterized protein	Up
Rv3699	3.27470687	http://www.uniprot.org/uniprot/O69667	Uncharacterized protein	Up
Rv1473	3.0910596	http://www.uniprot.org/uniprot/O53164	Probable macrolide-transport ATP-binding protein ABC transporter	Up
SseA	2.89811584	http://www.uniprot.org/uniprot/P9WHF7	Maf-like protein	Up
Rv3406	2.85041908	http://www.uniprot.org/uniprot/P9WKZ1	Oxidoreductase	Up
ino1	2.78947368	http://www.uniprot.org/uniprot/P9WKI1	Inositol-3-phosphate synthase	Up
gltD	2.7698665	http://www.uniprot.org/uniprot/P9WN19	Putative NADH-dependent glutamate synthase (small subunit)	Up
pks13	2.54393673	http://www.uniprot.org/uniprot/I6X8D2	4-Carboxymuconolactone decarboxylase	Up
mmaA4	0.50533896	http://www.uniprot.org/uniprot/Q79FX8	Hydroxymycolate synthase	Down
desA1	0.49308961	http://www.uniprot.org/uniprot/P9WNZ7	Acyl-ACP desaturase DesA	Down
Rv0248c	0.48947697	http://www.uniprot.org/uniprot/O53670	Fumarate reductase/succinate dehydrogenase flavoprotein subunit	Down
Rv0247c	0.48825875	http://www.uniprot.org/uniprot/O53669	Succinate dehydrogenase	Down
pncB2	0.48340731	http://www.uniprot.org/uniprot/P9WJI7	Nicotinic acid phosphoribosyltransferase PncB2	Down
fadD15	0.48100993	http://www.uniprot.org/uniprot/O53521	Long-chain fatty acid–CoA ligase FadD15, putative	Down
Rv1817	0.44241645	http://www.uniprot.org/uniprot/Q50616	3-Ketosteroid-delta-1-dehydrogenase	Down
hupB	0.40947969	http://www.uniprot.org/uniprot/P9WMK7	DNA-binding protein Hu	Down
fadD28	0.40220787	http://www.uniprot.org/uniprot/P9WQ59	Long-chain fatty acid–AMP ligase FadD28	Down
Rv1514c	0.37287415	http://www.uniprot.org/uniprot/P9WMX9	Glycosyltransferase	Down

Further KEGG analysis showed the top three pathways enriched in microbial metabolism in diverse environments, fatty acid metabolism, and citrate cycle (TCA cycle) (Figure 5C). For BCG, the genes related to microbial metabolism in diverse environments are upregulated, whereas six differential genes involved in TCA cycle were enriched including acn, Rv0247c, Rv0248c, KorA, Rv0794c, and SdhD; among them, the expression of Rv0247c, Rv0248c, KorA, and Rv0794c was increased in *M.tb*, whereas the expression of Acn and SdhD upregulated in BCG. TCA cycle pathway is known to produce energy, lipid, heme, and other precursors in *M.tb* (Tian et al., 2005). It suggests that *M.tb* in medium culture relies on TCA cycle more than BCG.

### Differential Protein Expression Between Intracellular and Extracellular *M.tb* During the Infection of Macrophages (*M.tb* In vs. *M.tb* Out)

**FIGURE 6:**
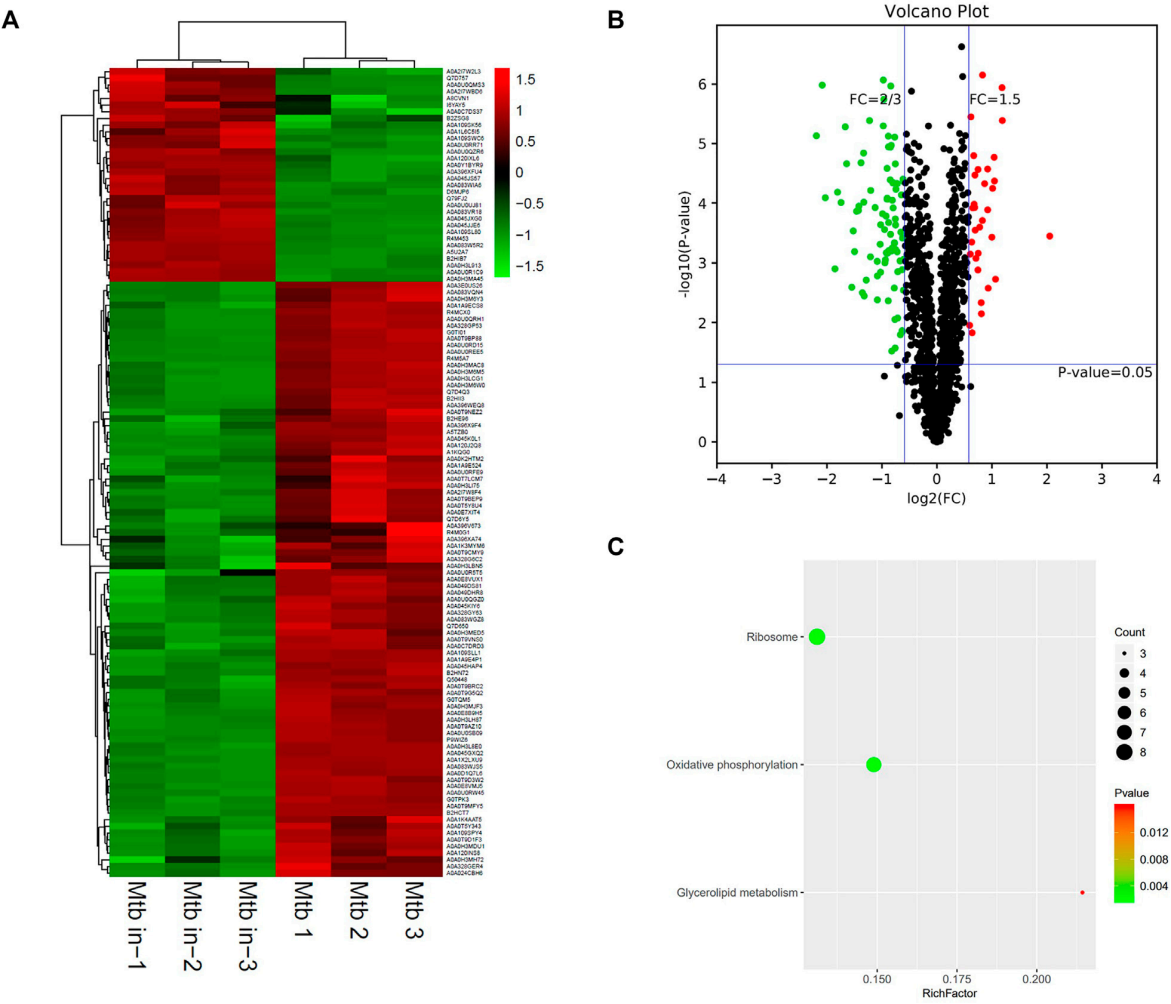
Different protein profile between intracellular *M.tb* and extracellular *M.tb*. **(A)** Heatmap of proteins differentially expressed between the group of intracellular *M.tb* and extracellular *M.tb*. Green-to-red scale indicates low-to-high proteins expression levels (*p* < 0.05). **(B)** Volcano plots of proteins differentially expressed between the group of intracellular *M.tb* and extracellular *M.tb*. The red dots on the right indicate the significantly upregulated proteins, whereas the downregulated are shown with green dots on the left. Differentially expressed proteins were defined based on a threshold fold change >1.5, at *p* < 0.05. **(C)** Top10 KEGG pathways (only three in this group) enriched for the significantly differentially expressed proteins. Entries with larger bubbles contain more differential genes. The color of the bubbles indicates the *p* value. The smaller the enrichment *p* value means the greater the degree of significance (*p* <0.05).

Based on the trusted protein selected, 121 differentiated proteins between *M.tb* in versus *M.tb* out were found (Figure 6A). The clustering heatmap between *M.tb* in and *M.tb* out showed that most (>70%) of the differential genes are repressed in *M.tb* in of macrophages compared with *M.tb* out (Figure 6B). The top 10 up-regulated proteins in intracellular *M.tb* compared with extracellular *M.tb* include Rv1498A, RpsF, Rv1405c, TB22.2, Rv1928c, LppE, Rv2466c, PPE41, FbpB, and Rv1674c. Rv1498A was the mostly up-regulated (four point one folds up), but the protein currently uncharacterized. RspF, 30S ribosomal protein S6, was modified by ATP-dependent glutamate ligase RimK and affected bacterium growth (Pipa et al., 2019). Therefore, it might play an important role in understanding the mechanism of *M.tb*. We also found that virulence-related gene PPE41 and RD region protein RV0223C were significantly up-regulated. When *M.tb* infected macrophages, PPE41 has been shown to induce macrophage necrosis (Tundup et al., 2014). Contrarily, the top 10 down-regulated proteins in intracellular *M.tb* compared with extracellular *M.tb* include HupB, NuoL, EmbA, GadB, MmpL13b, Rv0338c, AftC, QcrB, RpmF, and EccD3; among them, EccD3 was mostly down-regulated (five folds down) (Table 5). Eccd3, as part of the ESX-3 specialized secretion system, is important for iron and zinc uptake or homeostasis. RpmF, a 50S ribosomal protein, is down-regulated nearly fivefold too. In addition, of the top 10 proteins, many FadD family proteins are up-regulated after *M.tb* infection like FadE7, FadD7, FadD16, and FadD2, which might be relevant with *M.tb* virulence mechanism.

**TABLE 5:** The top10 differentially expressed proteins between intracellular *M.tb* and extracellular *M.tb.*

Genes	Fold change	UNIPROT_URL	Annotation	Differential expression
Rv1498A	4.140093396	http://www.uniprot.org/uniprot/I6XY36	Uncharacterized protein	Up
rpsF	2.279103053	http://www.uniprot.org/uniprot/P9WH31	30S ribosomal protein S6	Up
Rv1405c	2.26984127	http://www.uniprot.org/uniprot/P9WLY7	Putative methyltransferase	Up
TB22.2	2.091614333	http://www.uniprot.org/uniprot/I6YF08	hypothetical protein	Up
Rv1928c	2.063829787	http://www.uniprot.org/uniprot/P95286	Short-chain dehydrogenase	Up
lppE	2.016712009	http://www.uniprot.org/uniprot/O07750	lipoprotein LppE	Up
Rv2466c	1.998148148	http://www.uniprot.org/uniprot/O53193	Uncharacterized protein	Up
PPE41	1.907282133	http://www.uniprot.org/uniprot/Q79FE1	PPE family protein PPE41	Up
fbpB	1.894904459	http://www.uniprot.org/uniprot/P9WQP1	diacylglycerol acyltransferase/mycolyltransferase Ag85B	Up
Rv1674c	1.893905192	http://www.uniprot.org/uniprot/O53921	Transcriptional regulatory protein	Up
hupB	0.348717035	http://www.uniprot.org/uniprot/P9WMK7	DNA-binding protein Hu	Down
nuoL	0.342380423	http://www.uniprot.org/uniprot/P9WIW1	NADH dehydrogenase subunit	Down
embA	0.320856727	http://www.uniprot.org/uniprot/P9WNL9	Arabinosyltransferase EmbA	Down
gadB	0.315456165	http://www.uniprot.org/uniprot/I6YG46	Glutamate decarboxylase GadB	Down
mmpL13b	0.29913522	http://www.uniprot.org/uniprot/O06546	Trehalose monomycolate RND transporter MmpL3	Down
Rv0338c	0.286153846	http://www.uniprot.org/uniprot/O33268	Iron sulfur–binding protein	Down
aftC	0.276993265	http://www.uniprot.org/uniprot/P9WMZ7	α-(1->3)-Arabinofuranosyltransferase	Down
qcrB	0.245286344	http://www.uniprot.org/uniprot/P9WP37	Ubiquinol-cytochrome C reductase cytochrome subunit B	Down
rpmF	0.235881842	http://www.uniprot.org/uniprot/P9WH99	50S ribosomal protein	Down
eccD3	0.219337979	http://www.uniprot.org/uniprot/P9WNQ3	ESX-3 secretion system protein EccD	Down

KEGG pathway showed that differentiated genes were enriched on ribosome, oxidative phosphorylation, and fatty acid biosynthesis (Figure 6C). A highly dynamic intracellular environment and the *M.tb* are encountered with constant pressure; therefore, the intracellular bacteria need to coordinate its metabolic activity to adapt either replication or quiescence statue. The key feature is to generate ATP via oxidative phosphorylation. Many bacteria transited into hibernation of ribosome when faced with stresses to growth; the *M.tb* also has to rearrange ribosome pathway to adapt to the environment. In conclusion, *M.tb* survival strategies are linked with ATP generation and ribosome regulation to commensurate with intracellular environment.

### Differentiated Protein Expression Between Intracellular and Extracellular BCG During the Infection of Macrophages (BCG In vs. BCG Out)

**FIGURE 7:**
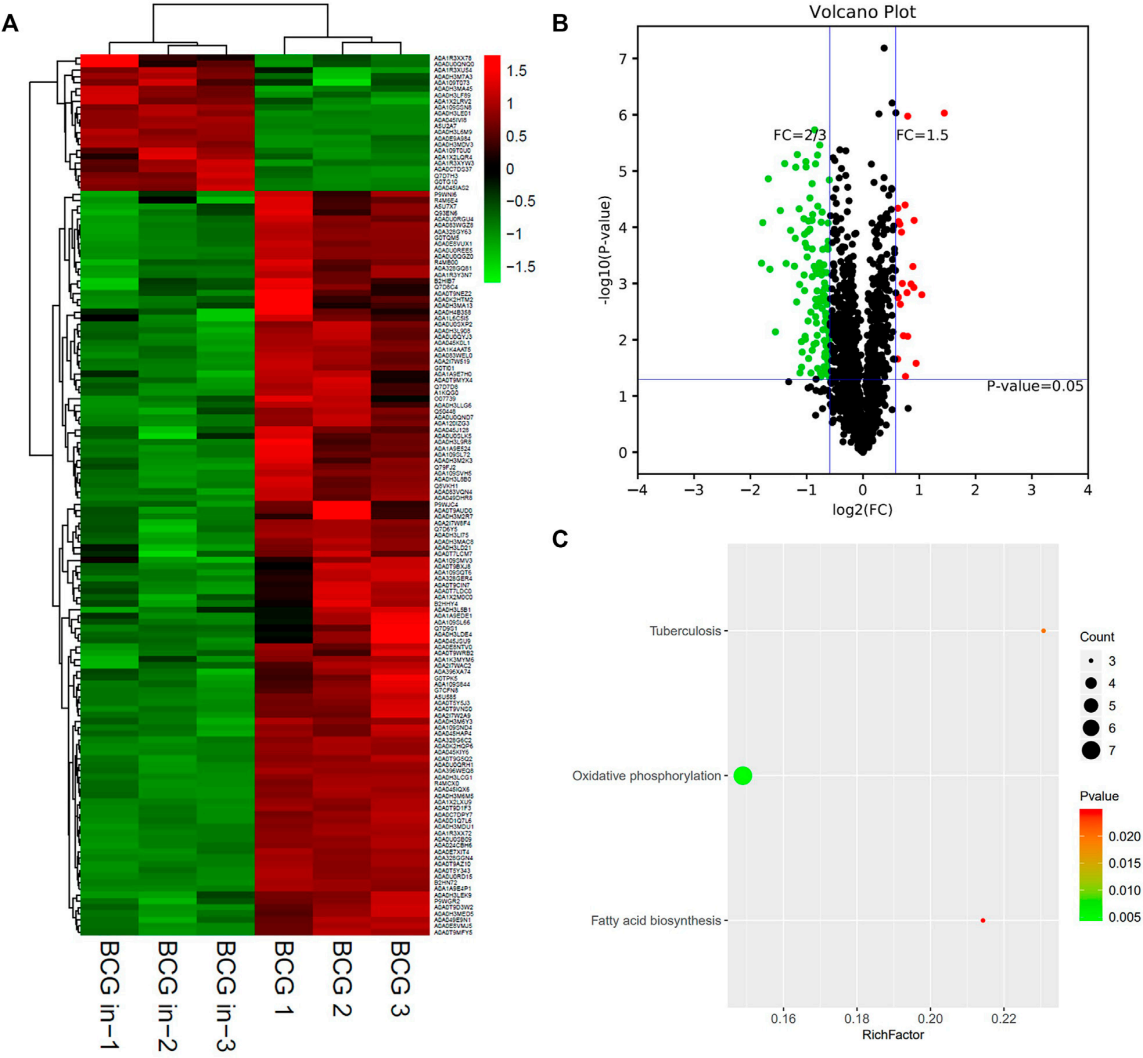
Different protein profile between intracellular BCG and extracellular BCG. **(A)** Heatmap of proteins differentially expressed between the group of intracellular BCG and extracellular BCG. Green-to-red scale indicates low-to-high proteins expression levels (*p* < 0.05). **(B)** Volcano plots of proteins differentially expressed between the group of intracellular BCG and extracellular BCG. The red dots on the right indicate the significantly upregulated proteins, whereas the downregulated are shown with green dots on the left. Differentially expressed proteins were defined based on a threshold fold change >1.5, at *p* < 0.05. **(C)** Top10 KEGG pathways (only three in this group) enriched for the significantly differentially expressed proteins. Entries with larger bubbles contain more differential genes. The color of the bubbles indicates the *p* value. The smaller the enrichment *p* value means the greater the degree of significance (*p* < 0.05).

Finally, we wanted to know how the expression profile of BCG, a vaccine strain, changed when BCG infects macrophages. Similar to intracellular *M.tb*, or even more trend was seen, most of the infected BCG’s proteins were inhibited compared with BCG control (Figure 7A). Both *M.tb* and BCG are silenced when it arrived in macrophages; this was consistent with the latent state of *M.tb* infection. In total, there were 142 differentiated proteins, with 22 proteins up-regulated and the remaining 120 proteins down-regulated between the group of BCG in and BCG out (Figure 7B). Furthermore, the detailed information of the top 10 is shown in Table 6. The top 10 up-regulated proteins in intracellular BCG compared with extracellular BCG include Rv1405c, Pks13, Rv1939, Rv3046c, RpsA, MtnP, MmsA, Rv0177, Rv0315, and Hsp. The top 1 protein of up-regulation is Rv1405c, with two-point sevenfold increasing. This protein is uncharacterized, and according to the Gene Ontology function annotations, it may have methyltransferase activity, hinting epigenetic modifications probably happened during the infection (Table 6). Another research showed Rv1405c might promote the intracellular survival of bacteria, but the detailed mechanism remains unknown (Healy et al., 2016). The top 10 down-regulated proteins in this group include EfpA, EmbA, MmpL7, RpmF, EccD5, EchA18, AftC, EccD3, QcrB, and NuoL. The NuoL is most significantly reduced protein among the list with the reduction three-point fivefold. It is a subunit of NDH-1 and may play a role in proton translocation. BCG oxidative activity counts more compared with *M.tb*. Besides, an unknown protein Rv1939 double its expression was identified, which is supposed to be a possible cobalamin synthetic protein (Supplementary Table S2). Of the top 10 proteins, LprG is an interesting protein up-regulated in intracellular BCG compared with extracellular BCG. LprG is a lipoprotein that modulates the host immune response against mycobacteria. Hovav et al. demonstrated that immunization with LprG induces a strong T_H_1 response, but Mariana Noelia Viale et al. found that the deletion of LprG can reduce the replication of *Mycobacterium avium* subspecies *paratuberculosis* in bovine macrophages. It seems a paradoxical mechanism but revealed that lprG is a key protein for *Mycobacterium* (Viale et al., 2021).

**TABLE 6:** The top10 differentially expressed proteins between intracellular BCG and extracellular BCG.

Genes	Fold change	UNIPROT_URL	Annotation	Differential expression
Rv1405c	2.721303732	http://www.uniprot.org/uniprot/P9WLY7	Methyltransferase	Up
pks13	2.063557858	http://www.uniprot.org/uniprot/I6X8D2	Polyketide synthase	Up
Rv1939	1.922230951	http://www.uniprot.org/uniprot/P95275	Oxidoreductase	Up
Rv3046c	1.877678224	http://www.uniprot.org/uniprot/I6YF16	Hypothetical protein	Up
rpsA	1.870317003	http://www.uniprot.org/uniprot/P9WH43	Ribosomal protein SA	Up
mtnP	1.848929421	http://www.uniprot.org/uniprot/O06401	S-methyl-5′-thioadenosine phosphorylase	Up
mmsA	1.807692308	http://www.uniprot.org/uniprot/O53816	Methylmalonate-semialdehyde dehydrogenase	Up
Rv0177	1.734368071	http://www.uniprot.org/uniprot/O07421	Mce associated protein	Up
Rv0315	1.734160241	http://www.uniprot.org/uniprot/O07242	β-1,3-Glucanase	Up
Hsp	1.719887955	http://www.uniprot.org/uniprot/O53673	Heat shock protein	Up
efpA	0.430492136	http://www.uniprot.org/uniprot/P9WJY5	MFS-type transporter EfpA	Down
embA	0.410486891	http://www.uniprot.org/uniprot/P9WNL9	Arabinosyltransferase A	Down
mmpL7	0.388711395	http://www.uniprot.org/uniprot/P9WJU7	Transmembrane transport protein MmpL7	Down
rpmF	0.38203724	http://www.uniprot.org/uniprot/P9WH99	50S ribosomal protein L32	Down
eccD5	0.361902625	http://www.uniprot.org/uniprot/P9WNP9	ESX-5 type VII secretion system protein EccD	Down
echA18	0.340576847	http://www.uniprot.org/uniprot/O50402	Enoyl-CoA hydratase	Down
aftC	0.318328667	http://www.uniprot.org/uniprot/P9WMZ7	α-(1->3)-Arabinofuranosyltransferase	Down
eccD3	0.312172088	http://www.uniprot.org/uniprot/P9WNQ3	ESX-3 secretion system protein EccD	Down
qcrB	0.291623037	http://www.uniprot.org/uniprot/P9WP37	Ubiquinol-cytochrome C reductase cytochrome subunit B	Down
nuoL	0.287162891	http://www.uniprot.org/uniprot/P9WIW1	NADH-quinone oxidoreductase subunit L	Down

Interestingly, KEGG pathway showed that oxidative phosphorylation was unique and most relevant in the group of BCG in vs. BCG, instead of both oxidative phosphorylation and ribosome in *M.tb* strains (Figure 7C). As the vaccine strain, BCG shares parts of the strategies with *M.tb* (containing the same genes in oxidative phosphorylation pathway: NuoL, QcrA, and QcrB), while it also lost other capacities to coordinate like ribosome pathway.

### Principal Component Analysis

**FIGURE 8:**
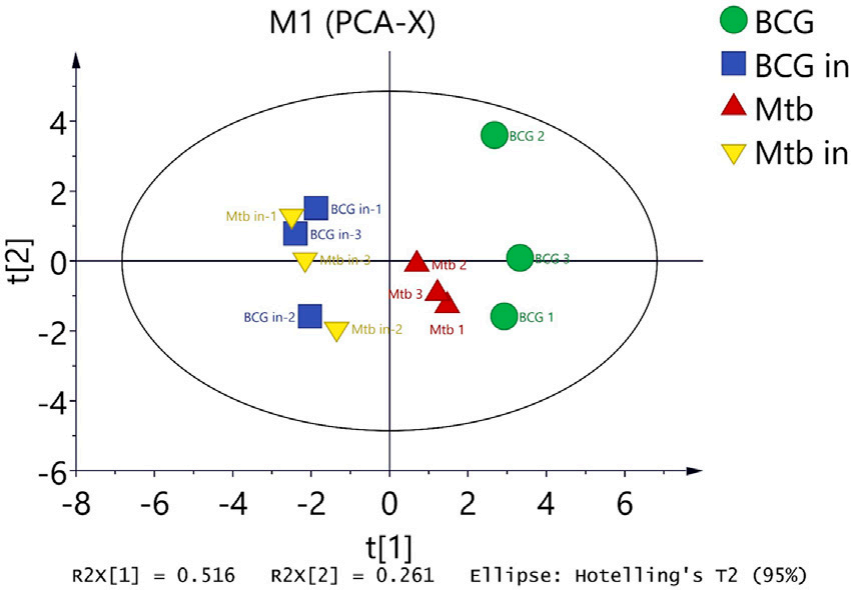
Differentiated protein expression with four groups were analyzed by principal component analysis (PCA). PCA displaying the distinct biological variation among BCG, BCG in, *M.tb*, and *M.tb* in samples. Each point in the figure represents a repetition in a grouping experiment. Different colors and shapes distinguish different groups. All samples are within 95% confidence intervals (Hotelling *T* ^2^ ellipse).

In summary, we could clearly show that BCG in and *M.tb* in clustered together, and BCG out and *M.tb* out clustered closer. But in between, there are clusters that form a separate group. At last, we used principal component analysis to summarize the different conditions. From the chart, we found the BCG was the farthest from the center; interestingly, the BCG in and *M.tb* in clustered as a whole separate group. *M.tb* group was close to both BCG and intracellular group but as a separate group (Figure 8). The information here showed that although *M.tb* and its vaccine strain have unique expression profiles, once they became intracellular bacteria (both BCG and *M.tb*) in macrophages, their protein expression were synchronized to the similar level.

## Discussion

Previously, there are several works on the differentiated proteins between *M.tb* and BCG targeting novel mechanism underlying virulence and pathogenesis focusing on the whole proteins or only membrane proteins. However, all works used the bacteria in medium culture. In current study, we compared the proteomics of both intracellular *M.tb* and BCG from infected macrophages and the extracellular *M.tb* and BCG grown in 7H9 medium. Therefore, the differential proteins including membrane proteins and whole proteins in this study revealed data more relevant to virulence of *M.tb*.

### Differential Proteins Common in Both Extracellular/Intracellular *M.tb* and BCG

Between *M.tb* in vs. *M.tb* out and BCG in vs. BCG out, there were 30 differential proteins up-regulated *M.tb* in vs. *M.tb* out, whereas 22 proteins in the group of BCG in vs. BCG out. However, only two proteins (Pks13 and Rv1405c) were commonly up-regulated, indicating both are essential for mycobacteria to enter THP-1 cells (Supplementary Table S3). Pks13 is required for mycolic acid biosynthesis; both *M.tb* and BCG need it to resist host immune cells (Wilson et al., 2013). Rv1405c is a methyltransferase that coordinates the adaptation of *M.tb* to the environment *in vivo* (Healy et al., 2016). On the other hand, there are both 41 differential proteins down-regulated in these two groups (*M.tb* in vs. *M.tb* out, BCG in vs. BCG out). Among them, 27 differential proteins were down-regulated commonly. Especially a series of oxidative phosphorylation pathway–related genes such as QcrA, QcrB, NuoL, NuoN, and CtaC that are closely related to energy metabolism were included. QcrA and QcrB are subunits of cytochrome-c complex, which is one of the transport electrons from the respiratory electron transport chain (Bahuguna et al., 2021). To establish an infection in the cells after invasion under the environment of phagosomes with low pH and oxygen and starvation, *M.tb* in and BCG in have to reduce their energy consumption and put themselves in a kind of dormant state.

The shared differential proteins between the group of BCG in vs. *M.tb* in and BCG out vs. *M.tb* out were further analyzed. There are 43 differential proteins up-regulated in group of BCG in vs. *M.tb* in, whereas there are 30 proteins up in group of BCG out vs. *M.tb* out. Among them, 20 proteins were commonly up-regulated. The phosphate-binding transporter lipoprotein PstS3 was a good immunogen inducing CD8(+) T-cell activation and both Th1 and Th17 immunity in mice (Palma et al., 2011). Hsp is up-regulated in the early stage of bacterial entry to cells and can help BCG stimulate the immune response of the hosts (Wilkinson et al., 2005). More interestingly, it seems that hsp is more important in BCG than *M.tb*, because it was only down-regulated after *M.tb* infection, but up-regulated in BCG in vs. BCG out, same as the two groups above. Thus, the results of the four groups were comprehensively analyzed, which provide partial explanation for *M.tb* survival better than BCG in host cells through immune evasion. In addition, Rv3699, Rv1762c, and Rv0825c are hypothetical proteins, which might be responsible for BCG immunogenicity and deserve to be further studied. On the other hand, there are 52 differential proteins down-regulated in group of BCG in vs. *M.tb* in, whereas there are 19 proteins down in group of BCG out vs. *M.tb* out. Among them, eight proteins were commonly down-regulated. A series of citrate cycle pathway–related genes, including Rv0247c and Rv0248c, are closely related to energy supply. In addition, some known virulence-related genes, such as RD region proteins and PPE family members, were not found in the shared differential proteins.

Taken together, among the differential proteins between BCG in/out and *M.tb* in/out, there are much more genes down-regulated than up-regulated for bacterial invasion of THP. Further, the down-regulation of genes is mainly enriched in pathways related to energy production. On the other hand, the commonly up-regulated genes are associated with induction of host immune response and bacterial intracellular survival.

### Differential Proteins Only Between Intracellular *M.tb* and BCG

There are 211 differentially expressed proteins found in total between *M.tb* in and BCG in. In order to better understand the virulence mechanism of *M.tb*, the four groups of the differential proteins were analyzed comprehensively, we found 41 differential proteins uniquely up-regulated in the intracellular persistence of *M.tb*, which are supposed to be associated with *M.tb* virulence. First, eight RD proteins Rv0223c, EspK, Rv2073c, EsxA, EpiA (Rv1512), EsxO, LpqG, and Rv1513 were identified; among them, Rv1512, Rv1513, and Rv2073c need to be investigated in the future. There are seven other known virulence-related proteins including PPE41, PPE51, VapC10, VapB19, PhoB1, PhoH2, and Eccb1. PPE41 has been shown to induce macrophage necrosis (Tundup et al., 2014). PPE51 participates in the uptake of disaccharides and acid growth arrest by tubercle bacilli (Baker and Abramovitch, 2018; Korycka-Machala et al., 2020). PhoB1 controlled SenX3–RegX3 activity in response to phosphate availability and regulating phosphate transport by the Pst system; besides, it also affected *M.tb* tolerance to antitubercular drugs (Brokaw et al., 2017). VapC10, VapB19, and PhoH2 belong to toxin–antitoxin system proteins, which can promote *M.tb* persistence (Gerdes and Maisonneuve, 2012; Kedzierska and Hayes, 2016). VapC10 is toxin, whereas VapB19 and PhoH2 are antitoxin. In response to stress conditions, the labile antitoxin is degraded, and toxin is released, which in turn halts transcription, translation, and so on, and that leads to growth inhibition and even cell death (Sala et al., 2014). PhoH2 with additional RNA helicase activity acts on specific RNA substrates and plays a role in the adaptive response to changing environmental conditions (Andrews and Arcus, 2015; Andrews and Arcus, 2020). Eccb1 is one of the conserved components that contracted the core structure of the ESX-1 system (Houben et al., 2012). Other interested proteins with the expression difference above fourfold contained TB22.2 (Rv3036c) and gnd1. TB22.2 was characterized as a novel cell wall–anchored esterase from *M.tb* that can efficiently hydrolyze soluble *p*-nitrophenyl esters (Chen et al., 2014). Gnd1 encoding 6-phosphogluconate dehydrogenase to engineering NADPH cofactor metabolism (Cheng et al., 2018). However, there are seven unknown function proteins including LppE, LpqD, Rv0795, Rv1532c, Rv2327, Rv3099c, and Rv1498A, which is worth to be investigated in the future.

For the differential proteins that are only up-regulated for the intracellular BCG, 23 proteins were identified, like Rv0315, FabG2, Rv1700, sigH, and Rv1523; they seem to be more related to immune response or fighting early stress in the host; only Rv1523 was related to virulence. Rv0315 is a novel dendritic cell maturation–inducing antigen that drives T-cell immune responses toward TH1 polarization and plays a crucial role in determining the nature of the immune response to TB (Korycka-Machala et al., 2020). Settles et al. found that FabG2-related vaccine candidates pgs1360 significantly reduced bacterial colonization in organs and induced an *M. paratuberculosis*–specific interferon γ production (Brokaw et al., 2017). Rv1700 and SigH play a key role in survival of bacteria against oxidative-stress (Raman et al., 2001; Patil et al., 2013). Methyltransferase Rv1523 induced virulence in mycobacteria (Ali et al., 2021).

In addition, there are six hypothetical proteins (Rv3046c, Rv0025, Rv1930c, Rv1262c, Rv0464c, and Rv0293c) and two less-information proteins (Rv0634c and Rv3842c) up-regulated in the group of intracellular BCG might be considered as candidate targets for vaccine research.

### Differential Proteins Only in Extracellular *M.tb* and BCG

There are 10 proteins just significantly up-regulated in BCG compared with *M.tb* are also mainly related to immunity and can stimulate the host’s immune response. Like Rv0494, an FadR homolog in *M.tb*, directly binds to long-chain fatty acyl-CoA to inhibit fatty acid synthase II regulation (Biswas et al., 2013). Rv2466c is a key oxidoreductase that mediates the reductive activation of TP053, a thienopyrimidine derivative that kills replicating and nonreplicating *M.tb* (Albesa-Jové et al., 2015). PPE26 is a TLR2 agonist that stimulates innate immunity and adaptive immunity (Su et al., 2015). Rv2052c and Rv3747 are hypothetical proteins that may also have good immunogenicity.

For the differential proteins that are only up-regulated for the extracellular *M.tb*, 11 proteins were identified. Three proteins are involved in fatty acid metabolism, fadA6, FadE19, and FadD28. FadD28 plays a key role in the biosynthesis of a phthiocerol dimycocerosate lipid that occurs only in the cell wall of pathogenic mycobacteria (Goyal et al., 2006). All of these three proteins are involved in the fatty metabolism pathway and are essential for the utilization of fat by *M.tb in vitro* (Goyal et al., 2006; Kawaji et al., 2010; Cox et al., 2019). This suggests that *M.tb* makes better use of fat.

Although this study conducted in-depth data mining on four groups of differentially compared proteins and found some interesting proteins through analysis, there are also many works that deserve further studies. The proteins found in this research are based only on THP-1 cell, so the further test verifications could be considered to identify with other macrophages or *in vivo*. In addition, for some significantly differential proteins, specific verification and functional analysis need to be verified by a series of subsequent experiments. These will help to provide reliable data for the screening of potential TB antigen for development of vaccine.

In conclusion, more than 70% of the proteins in both *M.tb* and BCG were down-regulated after they infected macrophages enriched mainly in energy production. The numbers of differentially expressed proteins between *M.tb* and BCG were significantly greater after infection than before infection. Forty-two proteins were identified to be up-regulated only in intracellular *M.tb* including eight RD proteins, whereas 22 up-regulated uniquely in intracellular BCG. Besides, only two proteins (Pks13 and Rv1405c) were commonly up-regulated for intracellular *M.tb* and BCG. Further, 10 differential unknown proteins were uniquely up-regulated in the intracellular *M.tb* (10) and BCG (8). These findings provide valuable data for further exploration of molecular mechanism for *M.tb* virulence and BCG immune response.

## Data Availability

The datasets presented in this study can be found in online repositories. The names of the repository/repositories and accession number(s) can be found in the article/Supplementary Material.

## References

[B1] Albesa-Jové D. Comino N. Tersa M. Mohorko E. Urresti S. Dainese E. (2015). The Redox State Regulates the Conformation of Rv2466c to Activate the Antitubercular Prodrug TP053. J. Biol. Chem. 290 (52), 31077–31089. 10.1074/jbc.m115.677039 26546681 PMC4692232

[B2] Ali S. Ehtram A. Arora N. Manjunath P. Roy D. Ehtesham N. Z. (2021). The *M. tuberculosis* Rv1523 Methyltransferase Promotes Drug Resistance through Methylation-Mediated Cell Wall Remodeling and Modulates Macrophages Immune Responses. Front. Cel Infect. Microbiol. 11, 622487. 10.3389/fcimb.2021.622487 PMC7994892 33777836

[B3] Andrews E. S. V. Arcus V. L. (2015). The Mycobacterial PhoH2 Proteins Are Type II Toxin Antitoxins Coupled to RNA Helicase Domains. Tuberculosis 95 (4), 385–394. 10.1016/j.tube.2015.03.013 25999286

[B4] Andrews E. S. V. Arcus V. L. (2020). PhoH2 Proteins Couple RNA Helicase and RNAse Activities. Protein Sci. 29 (4), 883–892. 10.1002/pro.3814 31886915 PMC7096712

[B5] Bahuguna A. Rawat S. Rawat D. S. (2021). QcrB in *Mycobacterium Tuberculosis* : The New Drug Target of Antitubercular Agents. Med. Res. Rev. 41 (4), 2565–2581. 10.1002/med.21779 33400275

[B6] Baker J. J. Abramovitch R. B. (2018). Genetic and Metabolic Regulation of *Mycobacterium Tuberculosis* Acid Growth Arrest. Sci. Rep. 8 (1), 4168. 10.1038/s41598-018-22343-4 29520087 PMC5843633

[B7] Beg A. Mathuria S. Athar F. Meena L. (2018). Structural and Functional Annotation of Rv1514c Gene of Mycobacterium Tuberculosis H 37 Rv as Glycosyl Transferases. J. Adv. Res. Biotech 3 (2), 1–9. 10.15226/2475-4714/3/2/00139

[B8] Biswas R. K. Dutta D. Tripathi A. Feng Y. Banerjee M. Singh B. N. (2013). Identification and Characterization of Rv0494: a Fatty Acid-Responsive Protein of the GntR/FadR Family from *Mycobacterium Tuberculosis* . Microbiology (Reading) 159 (Pt 5), 913–923. 10.1099/mic.0.066654-0 23475950

[B9] Brokaw A. M. Eide B. J. Muradian M. Boster J. M. Tischler A. D. (2017). Mycobacterium Smegmatis PhoU Proteins Have Overlapping Functions in Phosphate Signaling and Are Essential. Front. Microbiol. 8, 2523. 10.3389/fmicb.2017.02523 29326670 PMC5741670

[B10] Brosch R. Pym A. S. Gordon S. V. Cole S. T. (2001). The Evolution of Mycobacterial Pathogenicity: Clues from Comparative Genomics. Trends Microbiol. 9 (9), 452–458. 10.1016/s0966-842x(01)02131-x 11553458

[B11] Carranza C. Chavez-Galan L. (2019). Several Routes to the Same Destination: Inhibition of Phagosome-Lysosome Fusion by *Mycobacterium Tuberculosis* . Am. J. Med. Sci. 357 (3), 184–194. 10.1016/j.amjms.2018.12.003 30797501

[B12] Chen L. Dang G. Deng X. Cao J. Yu S. Wu D. (2014). Characterization of a Novel Exported Esterase Rv3036c from *Mycobacterium Tuberculosis* . Protein Expr. Purif. 104, 50–56. 10.1016/j.pep.2014.09.003 25224799

[B13] Cheng H. Wang S. Bilal M. Ge X. Zhang C. Fickers P. (2018). Identification, Characterization of Two NADPH-dependent Erythrose Reductases in the Yeast Yarrowia Lipolytica and Improvement of Erythritol Productivity Using Metabolic Engineering. Microb. Cel Fact 17, 133. 10.1186/s12934-018-0982-z PMC6114734 30157840

[B14] Cohen K. A. Manson A. L. Abeel T. Desjardins C. A. Chapman S. B. Hoffner S. (2019). Extensive Global Movement of Multidrug-Resistant *M. tuberculosis* Strains Revealed by Whole-Genome Analysis. Thorax 74 (9), 882–889. 10.1136/thoraxjnl-2018-211616 31048508 PMC6788793

[B15] Cox J. A. G. Taylor R. C. Brown A. K. Attoe S. Besra G. S. Fütterer K. (2019). Crystal Structure of *Mycobacterium Tuberculosis* FadB2 Implicated in Mycobacterial β-oxidation. Acta Cryst. Sect. D Struct. Biol. 75 (Pt 1), 101–108. 10.1107/S2059798318017242 30644849 PMC6333283

[B16] Dubaniewicz A. (2010). *Mycobacterium Tuberculosis* Heat Shock Proteins and Autoimmunity in Sarcoidosis. Autoimmun. Rev. 9 (6), 419–424. 10.1016/j.autrev.2009.11.015 19931650

[B17] Gerdes K. Maisonneuve E. (2012). Bacterial Persistence and Toxin-Antitoxin Loci. Annu. Rev. Microbiol. 6666, 103–123. 10.1146/annurev-micro-092611-150159 22994490

[B18] Goldberg M. F. Saini N. K. Porcelli S. A. (2014). Evasion of Innate and Adaptive Immunity by *Mycobacterium Tuberculosis* . Microbiol. Spectr. 2 (5), 2–5. 10.1128/microbiolspec.MGM2-0005-2013 26104343

[B19] Goyal A. Yousuf M. Rajakumara E. Arora P. Gokhale R. S. Sankaranarayanan R. (2006). Crystallization and Preliminary X-ray Crystallographic Studies of the N-Terminal Domain of FadD28, a Fatty-Acyl AMP Ligase fromMycobacterium Tuberculosis. Acta Cryst. Sect. F 62 (Pt 4), 350–352. 10.1107/S1744309106005938 PMC2222565 16582482

[B20] Healy C. Golby P. MacHugh D. E. Gordon S. V. (2016). The MarR Family Transcription Factor Rv1404 Coordinates Adaptation of *Mycobacterium Tuberculosis* to Acid Stress via Controlled Expression of Rv1405c, a Virulence-Associated Methyltransferase. Tuberculosis 97, 154–162. 10.1016/j.tube.2015.10.003 26615221

[B21] Houben E. N. G. Bestebroer J. Ummels R. Wilson L. Piersma S. R. Jiménez C. R. (2012). Composition of the Type VII Secretion System Membrane Complex. Mol. Microbiol. 86 (2), 472–484. 10.1111/j.1365-2958.2012.08206.x 22925462

[B22] Kawaji S. Zhong L. Whittington R. J. (2010). Partial Proteome of *Mycobacterium avium* Subsp. Paratuberculosis under Oxidative and Nitrosative Stress. Vet. Microbiol. 145 (3-4), 252–264. 10.1016/j.vetmic.2010.03.025 20413229

[B23] Kedzierska B. Hayes F. (2016). Emerging Roles of Toxin-Antitoxin Modules in Bacterial Pathogenesis. Molecules 21 (6), 790. 10.3390/molecules21060790 PMC6273597 27322231

[B24] Korycka-Machala M. Pawelczyk J. Borowka P. Dziadek B. Brzostek A. Kawka M. (2020). PPE51 Is Involved in the Uptake of Disaccharides by *Mycobacterium Tuberculosis* . Cells-Basel 9 (3), 603. 10.3390/cells9030603 PMC7140425 32138343

[B25] Li M. Cui J. Niu W. Huang J. Feng T. Sun B. (2019). Long Non-coding PCED1B-AS1 Regulates Macrophage Apoptosis and Autophagy by Sponging miR-155 in Active Tuberculosis. Biochem. Biophys. Res. Commun. 509 (3), 803–809. 10.1016/j.bbrc.2019.01.005 30621915

[B26] Liu C. H. Liu H. Ge B. (2017). Innate Immunity in Tuberculosis: Host Defense vs Pathogen Evasion. Cell Mol. Immunol. 14 (12), 963–975. 10.1038/cmi.2017.88 28890547 PMC5719146

[B27] Palma C. Spallek R. Piccaro G. Pardini M. Jonas F. Oehlmann W. (2011). The *M. tuberculosis* Phosphate-Binding Lipoproteins PstS1 and PstS3 Induce Th1 and Th17 Responses that Are Not Associated with protection against *M. tuberculosis* Infection. Clin. Develop. Immunol. 2011, 690328. 10.1155/2011/690328 PMC3095447 21603219

[B28] Patil A. G. G. Sang P. B. Govindan A. Varshney U. (2013). *Mycobacterium Tuberculosis* MutT1 (Rv2985) and ADPRase (Rv1700) Proteins Constitute a Two-Stage Mechanism of 8-Oxo-dGTP and 8-Oxo-GTP Detoxification and Adenosine to Cytidine Mutation Avoidance. J. Biol. Chem. 288 (16), 11252–11262. 10.1074/jbc.M112.442566 23463507 PMC3630869

[B29] Pipa B. Mvn B. Mpra B. Mvsa B. Kd A. Iaoa B. (2019). Oligoglutamylation of E.Coli Ribosomal Protein S6 Is under Growth Phase Control. Biochimie 167 (C), 61–67. 10.1016/j.biochi.2019.09.008 31520657

[B30] Raman S. Song T. Puyang X. Bardarov S. Jacobs W. R. Husson R. N. (2001). The Alternative Sigma Factor SigH Regulates Major Components of Oxidative and Heat Stress Responses in *Mycobacterium Tuberculosis* . J. Bacteriol. 183 (20), 6119–6125. 10.1128/Jb.183.20.6119-6125.2001 11567012 PMC99691

[B31] Sala A. Bordes P. Genevaux P. (2014). Multiple Toxin-Antitoxin Systems in *Mycobacterium Tuberculosis* . Toxins 6 (3), 1002–1020. 10.3390/toxins6031002 24662523 PMC3968373

[B32] Su H. Kong C. Zhu L. Huang Q. Luo L. Wang H. (2015). PPE26 Induces TLR2-dependent Activation of Macrophages and Drives Th1-type T-Cell Immunity by Triggering the Cross-Talk of Multiple Pathways Involved in the Host Response. Oncotarget 6 (36), 38517–38537. 10.18632/oncotarget.5956 26439698 PMC4770718

[B33] Tanghe A. Lefèvre P. Denis O. D'Souza S. Braibant M. Lozes E. (1999). Immunogenicity and Protective Efficacy of Tuberculosis DNA Vaccines Encoding Putative Phosphate Transport Receptors. J. Immunol. 162 (2), 1113–1119. 9916741

[B34] Tian J. Bryk R. Itoh M. Suematsu M. Nathan C. (2005). Variant Tricarboxylic Acid Cycle in *Mycobacterium Tuberculosis*: Identification of -ketoglutarate Decarboxylase. Proc. Natl. Acad. Sci. 102 (30), 10670–10675. 10.1073/pnas.0501605102 16027371 PMC1180764

[B35] Trivedi O. A. Arora P. Vats A. Ansari M. Z. Tickoo R. Sridharan V. (2005). Dissecting the Mechanism and Assembly of a Complex Virulence Mycobacterial Lipid. Mol. Cel 17 (5), 631–643. 10.1016/j.molcel.2005.02.009 15749014

[B36] Tundup S. Mohareer K. Hasnain S. E. (2014). Mycobacterium tuberculosisPE25/PPE41 Protein Complex Induces Necrosis in Macrophages: Role in Virulence and Disease Reactivation? FEBS Open Bio 4, 822–828. 10.1016/j.fob.2014.09.001 PMC4219985 25379378

[B37] Viale M. N. Colombatti Olivieri M. A. Alonso N. Moyano R. D. Imperiale B. Morcillo N. (2021). Effect of the Deletion of lprG and P55 Genes in the K10 Strain of *Mycobacterium avium* Subspecies Paratuberculosis. Res. Vet. Sci. 138, 1–10. 10.1016/j.rvsc.2021.05.019 34087563

[B38] Wang L. Wu J. Li J. Yang H. Tang T. Liang H. (2020). Host-mediated Ubiquitination of a Mycobacterial Protein Suppresses Immunity. Nature 577 (7792), 682–688. 10.1038/s41586-019-1915-7 31942069

[B39] Weiss G. Schaible U. E. (2015). Macrophage Defense Mechanisms against Intracellular Bacteria. Immunol. Rev. 264 (1), 182–203. 10.1111/imr.12266 25703560 PMC4368383

[B40] Wilkinson K. A. Stewart G. R. Newton S. M. Vordermeier H. M. Wain J. R. Murphy H. N. (2005). Infection Biology of a Novel α-Crystallin ofMycobacterium Tuberculosis: Acr2. J. Immunol. 174 (7), 4237–4243. 10.4049/jimmunol.174.7.4237 15778386

[B41] Wilson R. Kumar P. Parashar V. Vilchèze C. Veyron-Churlet R. Freundlich J. S. (2013). Antituberculosis Thiophenes Define a Requirement for Pks13 in Mycolic Acid Biosynthesis. Nat. Chem. Biol. 9 (8), 499–506. 10.1038/nchembio.1277 23770708 PMC3720791

[B42] Xiong X. Wang R. Deng D. Chen Y. Liu H. Wang T. (2017). Comparative Genomics of a Bovine *Mycobacterium Tuberculosis* Isolate and Other Strains Reveals its Potential Mechanism of Bovine Adaptation. Front. Microbiol. 8, 2500. 10.3389/fmicb.2017.02500 29312206 PMC5733104

[B43] Zhao Y. Wang Z. Zhang W. Zhang L. (2019). MicroRNAs Play an Essential Role in Autophagy Regulation in Various Disease Phenotypes. Biofactors 45, 844–856. 10.1002/biof.1555 31418958 PMC6916288

[B44] Zhu B. Dockrell H. M. Ottenhoff T. H. M. Evans T. G. Zhang Y. (2018). Tuberculosis Vaccines: Opportunities and Challenges. Respirology 23 (4), 359–368. 10.1111/resp.13245 29341430

