# Supplementary material for: Comparative Analysis on Proteomics Profiles of Intracellular and Extracellular M.tb and BCG From Infected Human Macrophages
Source: Front Genet. 2022 Mar 28;13:847838. doi: 10.3389/fgene.2022.847838 (PMC8995892; doi:10.3389/fgene.2022.847838)
Supplement: Supplementary file 1 [file DataSheet1.zip › Suppl Table 2- 4.DOCX]

**S Table 2 Unknown differentially expressed proteins among the four groups**

| **Group** | **Gene** | **FoldChange** | **P-value** | **Differential expression** | **Description** |
| --- | --- | --- | --- | --- | --- |
| BCG-MTB | Rv1358 | 4.58123371 | 0.000458 | up | Transcriptional regulator |
|  | Rv3699 | 3.274706868 | 0.000224 | up | Uncharacterized |
| MTB in-MTB | Rv1498A | 4.140093396 | 0.000356 | up | Uncharacterized |
|  | Rv1928c | 2.063829787 | 4.25E-05 | up | short-chain type dehydrogenase/reductase |
|  | lppE | 2.016712009 | 5.64E-05 | up | Lipoprotein |
|  | RV1674C | 1.893905192 | 2.68E-05 | up | Transcriptional regulator |
| BCG in-BCG | Rv1939 | 1.922230951 | 0.026247 | up | Oxidoreductase |
|  | Rv3046c | 1.877678224 | 7.55E-05 | up | Uncharacterized |
| MTB in-BCG in | Rv2588c | 3.295882763 | 0.046107 | up | Uncharacterized |
|  | Rv1762c | 3.11299804 | 0.000469 | up | Uncharacterized |
|  | Rv0025 | 2.979821958 | 8.84E-05 | up | Uncharacterized |
|  | lppE | 0.369435344 | 5.68E-05 | down | Lipoprotein |
|  | pncB2 | 0.349864499 | 1.23E-05 | down | Functional Nicotinate Phosphoribosyltransferases |
|  | Rv1498A | 0.325813729 | 0.000498 | down | Uncharacterized |
|  | vapB19 | 0.308091531 | 0.006458 | down | Antitoxin VapB19 |
|  | Rv0795 | 0.277755018 | 0.000394 | down | Uncharacterized |
|  | Rv1817 | 0.270431497 | 0.001101 | down | Flavoprotein |

**S Table 3 Differential proteins in *M.tb* in vs *M.tb* out and BCG in vs BCG out**

| *M.tb* in vs *M.tb* | FoldChange | BCG in vs BCG | FoldChange |
| --- | --- | --- | --- |
| Rv1498A | 4.140093396 | Rv1405c | 2.721303732 |
| rpsF | 2.279103053 | pks13 | 2.063557858 |
| Rv1405c | 2.26984127 | Rv1939 | 1.922230951 |
| TB22.2 | 2.091614333 | Rv3046c | 1.877678224 |
| Rv1928c | 2.063829787 | rpsA | 1.870317003 |
| lppE | 2.016712009 | mtnP | 1.848929421 |
| Rv2466c | 1.998148148 | mmsA | 1.807692308 |
| PPE41 | 1.907282133 | Rv0177 | 1.734368071 |
| fbpB | 1.894904459 | Rv0315 | 1.734160241 |
| Rv1674c | 1.893905192 | hsp | 1.719887955 |
| lpqD | 1.823163622 | Rv1060 | 1.687558907 |
| apa | 1.776012301 | sigH | 1.679931973 |
| add | 1.773097176 | Rv0025 | 1.644611857 |
| pks13 | 1.751318102 | Rv2132 | 1.622937294 |
| fadD7 | 1.747022121 | trxB1 | 1.606592466 |
| gnd1 | 1.71377749 | Rv0898c | 1.583668544 |
| fadD16 | 1.682451253 | lprG | 1.575217886 |
| Rv0223c | 1.678754434 | fadE21 | 1.55026455 |
| fbpC | 1.67557652 | Rv1473 | 1.544724156 |
| mce1B | 1.634831461 | Rv1700 | 1.538817006 |
| glpQ1 | 1.61987041 | Rv0347 | 1.536821705 |
| Rv2017 | 1.609368285 | Rv0575c | 1.533082707 |
| Rv1463 | 1.597809856 |  |  |
| lprA | 1.589658966 |  |  |
| fadD2 | 1.569959603 |  |  |
| dsbF | 1.558704453 |  |  |
| katG | 1.550709939 |  |  |
| rplF | 1.534442724 |  |  |
| Rv2406c | 1.522718293 |  |  |
| fadE7 | 1.510090167 |  |  |
| mmpL7 | 0.535014 | qcrA | 0.53515398 |
| Rv2520c | 0.534673 | lysA | 0.534722222 |
| eccC5 | 0.533896 | Rv0461 | 0.527725118 |
| rip3 | 0.533045 | sdhD | 0.526811118 |
| ppiA | 0.528801 | Rv3645 | 0.520995725 |
| Rv2969c | 0.527215 | ppsB | 0.520450649 |
| embC | 0.522774 | Rv2520c | 0.519875776 |
| Rv3850 | 0.51965 | epiA | 0.512605042 |
| rplR | 0.51256913 | Rv0412c | 0.511835013 |
| ctaC | 0.509237875 | ftsK | 0.50879567 |
| rpsT | 0.509021601 | rplT | 0.507045617 |
| rplV | 0.504085338 | Rv3193c | 0.503341902 |
| nuoN | 0.492003763 | Rv0338c | 0.500670691 |
| Rv1747 | 0.489393939 | ctaC | 0.496986301 |
| htrA | 0.472752357 | guaB | 0.49586587 |
| Rv3645 | 0.47240782 | Rv0913c | 0.492548253 |
| Rv0412c | 0.471618037 | nuoN | 0.491695228 |
| rplT | 0.459230769 | ppsD | 0.490649296 |
| qcrA | 0.434429731 | Rv1707 | 0.487777778 |
| Rv1226c | 0.433131802 | rip3 | 0.487544484 |
| lysA | 0.42817995 | Rv0225 | 0.484743562 |
| ftsK | 0.410447761 | embC | 0.483503154 |
| Rv2536 | 0.400451097 | Rv3492c | 0.473596132 |
| ctpC | 0.398311897 | Rv1534 | 0.470038733 |
| Rv3193c | 0.397229162 | Rv3728 | 0.469002165 |
| fdxC | 0.391058291 | Rv0790c | 0.460704607 |
| hsp * | 0.384694933 | Rv0494 | 0.458825927 |
| efpA | 0.374378916 | mbtE | 0.456563442 |
| guaB | 0.372186093 | Rv2536 | 0.442366732 |
| eccD5 | 0.363531846 | gadB | 0.437701396 |
| Rv1707 | 0.354463922 | mmpL13b | 0.435037274 |
| hup | 0.348717035 | efpA | 0.430492136 |
| nuoL | 0.342380423 | embA | 0.410486891 |
| embA | 0.320856727 | mmpL7 | 0.388711395 |
| gadB | 0.315456165 | rpmF | 0.38203724 |
| mmpL13b | 0.29913522 | eccD5 | 0.361902625 |
| Rv0338c | 0.286153846 | echA18 | 0.340576847 |
| aftC | 0.276993265 | aftC | 0.318328667 |
| qcrB | 0.245286344 | eccD3 | 0.312172088 |
| rpmF | 0.235881842 | qcrB | 0.291623037 |
| eccD3 | 0.219337979 | nuoL | 0.287162891 |

**S Table 4 Differential proteins in BCG in vs *M.tb* in and BCG out vs *M.tb* out**

| BCG in vs *M.tb* in | FoldChange | BCG out vs *M.tb* out | FoldChange |
| --- | --- | --- | --- |
| rpsA | 13.94335938 | rpsA | 7.17481203 |
| fadD29 | 4.713255185 | fadD29 | 4.75609756 |
| Rv1473 | 4.471317829 | Rv1358 | 4.58123371 |
| Rv3699 | 3.954741379 | Rv3699 | 3.27470687 |
| **sseA** | 3.82406015 | Rv1473 | 3.0910596 |
| Rv1358 | 3.421887391 | **sseA** | 2.89811584 |
| Rv2588c | 3.295882763 | Rv3406 | 2.85041908 |
| Rv1762c | 3.112998041 | ino1 | 2.78947368 |
| pks13 | 2.997491219 | gltD | 2.7698665 |
| Rv0025 | 2.979821958 | pks13 | 2.54393673 |
| Rv1700 | 2.776542524 | Rv1762c | 2.48929664 |
| Rv0825c | 2.708517699 | Rv3492c | 2.48177204 |
| gltD | 2.686095932 | Rv0825c | 2.21493625 |
| Rv0315 | 2.544515494 | Rv2466c | 2.19197531 |
| **prpR** | 2.499433749 | **acn** | 2.15729266 |
| **acn** | 2.41613723 | **prpR** | 2.12788823 |
| opcA | 2.395221503 | amiC | 2.11686233 |
| Rv1719 | 2.370133333 | Rv0494 | 2.11192424 |
| **pstS3** | 2.363636364 | Rv1458c | 2.09209864 |
| Rv1523 | 2.323264441 | aldA | 2.06246634 |
| **hsp** | 2.287634409 | **hsp** | 2.06209386 |
| amiC | 2.270844397 | Rv3728 | 2.04220257 |
| glpQ1 | 2.254237288 | Rv1719 | 2.02771855 |
| sigH | 2.241942805 | Rv1178 | 2.01225837 |
| Rv1939 | 2.187751453 | PPE26 | 2.00951475 |
| lldD | 2.150342801 | Rv2423 | 1.93825374 |
| Rv1178 | 2.128834356 | sirR | 1.93115942 |
| moeB2 | 2.110429448 | Rv2052c | 1.916969 |
| nrdF2 | 2.100461302 | Rv3747 | 1.879293 |
| Rv1930c | 2.083698297 | **pstS3** | 1.857621 |
| Rv1262c | 2.077179609 |  |  |
| Rv0464c | 2.073009892 |  |  |
| Rv0293c | 2.070718878 |  |  |
| pmmB | 2.056451613 |  |  |
| Rv3046c | 2.025210084 |  |  |
| mycP5 | 2.024284943 |  |  |
| Rv3406 | 2.021052632 |  |  |
| Rv1405c | 2.014335664 |  |  |
| fabG2 | 2.008161306 |  |  |
| subI | 2.005641026 |  |  |
| Rv2423 | 1.929102628 |  |  |
| ino1 | 1.921606119 |  |  |
| Rv0634c | 1.921401515 |  |  |
| lprA | 0.519031142 | fadA6 | 0.557442793 |
| eccCb1 | 0.516767455 | secE2 | 0.554530657 |
| cyp125 | 0.510622887 | hbhA | 0.552547771 |
| Rv2327 | 0.510451613 | Rv2956 | 0.52744511 |
| Rv0223c | 0.510448462 | narH | 0.517479499 |
| espK | 0.509849663 | narG | 0.51582868 |
| mce2F | 0.500747012 | Rv0338c | 0.50974359 |
| phoU1* | 0.499334221 | fadE19 | 0.50796422 |
| Rv2073c | 0.497898056 | nuoF | 0.5053396 |
| Rv0060 | 0.496320911 | mmaA4 | 0.50533896 |
| phoH2 | 0.495876563 | desA1 | 0.49308961 |
| lpqD | 0.49562993 | Rv0248c | 0.48947697 |
| PPE51 | 0.494120941 | Rv0247c | 0.48825875 |
| Rv1928c | 0.477955692 | pncB2 | 0.48340731 |
| esxA | 0.473671138 | **fadD15** | 0.48100993 |
| epiA | 0.473275862 | Rv1817 | 0.44241645 |
| Rv1751 | 0.470767666 | hup | 0.40947969 |
| prpC | 0.469735504 | fadD28 | 0.40220787 |
| Rv3888c | 0.46860525 | Rv1514c | 0.37287415 |
| pstB1 | 0.46775427 |  |  |
| esxO | 0.458614654 |  |  |
| narH | 0.453774137 |  |  |
| Rv0794c | 0.453357721 |  |  |
| lpqG | 0.448402948 |  |  |
| Rv1191 | 0.444208852 |  |  |
| vapC10 | 0.440315315 |  |  |
| tuf | 0.436162709 |  |  |
| Rv2956 | 0.429717899 |  |  |
| PPE41 | 0.428541623 |  |  |
| crp | 0.428019104 |  |  |
| fbpB | 0.427941176 |  |  |
| pstB2 | 0.426829268 |  |  |
| Rv1532c | 0.425759417 |  |  |
| Rv1513 | 0.425718461 |  |  |
| rpsF | 0.412602051 |  |  |
| Rv0120c | 0.412592416 |  |  |
| **fadD15** | 0.405745554 |  |  |
| katG | 0.405493787 |  |  |
| Rv0575c | 0.387664217 |  |  |
| mmaA3 | 0.383870968 |  |  |
| Rv0248c | 0.38104724 |  |  |
| desA1 | 0.379489849 |  |  |
| lppE Rv1881c | 0.369435344 |  |  |
| Rv3099c | 0.364488171 |  |  |
| pncB2 | 0.349864499 |  |  |
| Rv1498A | 0.325813729 |  |  |
| vapB19 | 0.308091531 |  |  |
| **pstS1** | 0.293881008 |  |  |
| Rv0795 | 0.277755018 |  |  |
| Rv1817 | 0.270431497 |  |  |
| gnd1 | 0.251934327 |  |  |
| TB22.2 | 0.233486401 |  |  |
